# Differences in peripheral sensory input to the olfactory bulb between male and female mice

**DOI:** 10.1038/srep45851

**Published:** 2017-04-26

**Authors:** Marley D. Kass, Lindsey A. Czarnecki, Andrew H. Moberly, John P. McGann

**Affiliations:** 1 Behavioral & Systems Neuroscience Section, Department of Psychology Rutgers, The State University of New Jersey, 152 Frelinghuysen Road, Piscataway, NJ 08854, USA.

## Abstract

Female mammals generally have a superior sense of smell than males, but the biological basis of this difference is unknown. Here, we demonstrate sexually dimorphic neural coding of odorants by olfactory sensory neurons (OSNs), primary sensory neurons that physically contact odor molecules in the nose and provide the initial sensory input to the brain’s olfactory bulb. We performed *in vivo* optical neurophysiology to visualize odorant-evoked OSN synaptic output into olfactory bub glomeruli in unmanipulated (gonad-intact) adult mice from both sexes, and found that in females odorant presentation evoked more rapid OSN signaling over a broader range of OSNs than in males. These spatiotemporal differences enhanced the contrast between the neural representations of chemically related odorants in females compared to males during stimulus presentation. Removing circulating sex hormones makes these signals slower and less discriminable in females, while in males they become faster and more discriminable, suggesting opposite roles for gonadal hormones in influencing male and female olfactory function. These results demonstrate that the famous sex difference in olfactory abilities likely originates in the primary sensory neurons, and suggest that hormonal modulation of the peripheral olfactory system could underlie differences in how males and females experience the olfactory world.

Though sex differences in olfaction can be complicated by factors like sensory experience^1^,^2^, age^3^,^4^,^5^, and stimulus identity^5^,^6^,^7^,^8^, the female olfactory system is generally more effective than the male olfactory system^9^. Females tend to exhibit enhanced sensitivity to odors^6^,^10^,^11^,^12^,^13^,^14^ as well as better discrimination and identification abilities^15^,^16^ than males.

The biological basis of these sensory differences could be partly related to differences in endocrine status. Experimentally-induced alterations in hormonal status can influence odor detection thresholds in males^13^,^17^, and olfactory sensitivity in females can also vary with fluctuations in sex hormones^18^,^19^,^20^,^21^. Olfactory dysfunction can develop in women during menopause, and these deficits can be ameliorated by sex hormone therapy^22^,^23^. Analogously, 17β-estradiol treatment in gonadectomized mice enhances the retention of an odor memory^24^ and also ameliorates olfactotoxicant-induced discrimination deficits^25^.

Despite the clear linkage between sex hormones and olfaction, it has been difficult to determine where in the olfactory system these hormones are acting. A growing body of work shows that peripheral chemosensory signaling can be modified by experience and internal state^2^,^26^,^27^,^28^, as well as by the activity of structures that process multisensory information^29^. The main olfactory epithelium expresses α- and β-type estrogen receptors^30^, and estrogen replacement can protect against olfactory sensory neuron (OSN) loss in gonadectomized mice^31^,^32^, suggesting that sex and hormone effects could have a peripheral locus. Similarly, the vomeronasal organ undergoes state-dependent filtering in which sex hormones influence the responses of peripheral sensory neurons to specific chemical cues and consequently also behavioral responses to those cues^33^,^34^. We thus hypothesized that sex differences in olfaction could be at least partly mediated by sexually dimorphic sensory processing in the peripheral olfactory system.

Olfactory transduction occurs in the olfactory epithelium, where odorant molecules stimulate neural activity by binding to G protein-coupled odor receptors in the cilia of OSNs. The OSNs project their axons to the brain’s olfactory bulb, where they segregate by receptor type, such that all the OSNs expressing a given receptor converge into one or two glomeruli on the surface of the olfactory bulb. Because each OSN expresses only one out of a large repertoire of odor receptors, a given odorant activates only a small subset of OSNs and thus drives neural input to a corresponding subset of glomeruli in the olfactory bulb. The brain’s initial neural code for the identity of an odorant in the nose is thus the spatiotemporal pattern of olfactory bulb glomeruli receiving synaptic input from OSNs. These patterns of odorant-evoked neurotransmitter release from OSN axon terminals in the olfactory bulb can be visualized in a line of gene-targeted mice that express the fluorescent exocytosis indicator synaptopHluorin (spH) under the control of the olfactory marker protein (OMP) promoter^35^. We performed *in vivo* optical neurophysiology to visualize these spatiotemporal patterns in gonad-intact, adult OMP-spH mice of both sexes to see if there was a sex difference in odor representations at the initial sensory input to the brain. We then tested whether circulating sex hormones influenced these initial neural representations of odors by performing the same *in vivo* optical imaging procedures in control and gonadectomized mice of both sexes.

## Results

### Odors evoke OSN input to more olfactory bulb glomeruli in females than in males

Gonad-intact male (*N* = 8) and female (*N* = 11) OMP-spH mice were each implanted with a bilateral cranial window overlying the dorsal surface of the olfactory bulbs. Subjects remained anesthetized while the synaptic output of OSNs was visualized by fluorescence microscopy during 4 6-sec presentations of each stimulus in the odor panel, which consisted of 3 separate concentrations of 4 monomolecular odorants. Because spH is a cumulative measure of exocytosis from OSNs, these long presentations permit odor-evoked responses to be detected even in weakly activated OSN populations^35^. The number of olfactory bulb glomeruli exhibiting a measurable odorant-evoked fluorescence response was quantified for each odorant in the panel in each mouse. These numbers of odorant-evoked glomerular responses were then analyzed via a mixed, 3-way ANOVA to evaluate potential sex differences in the number of olfactory bulb glomeruli receiving OSN input.

**Figure 1:**
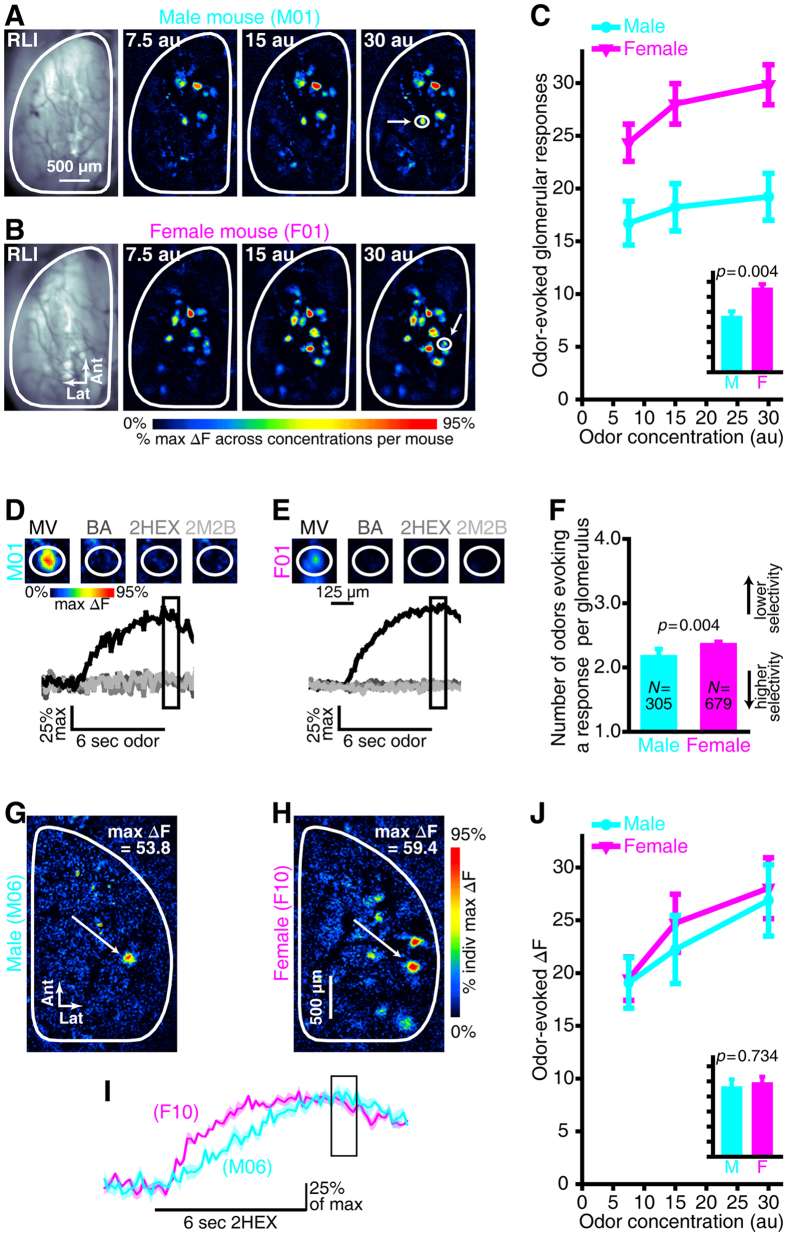
Odorant-evoked glomerular response maps contain a greater number of glomeruli receiving synaptic input in unmanipulated females than in unmanipulated males. (**A**,**B**) Resting light intensity (RLI) images through the cranial window and pseudocolored difference maps showing the peak responses evoked by 3 concentrations of MV from representative male (**A**), M01) and female (**B**), F01) subjects. The circled callouts indicate the example glomeruli in (**D**,**E)**. (**C**) Mean ± SEM number of odorant-evoked glomerular responses plotted as a function of odorant concentration. The inset shows the main effect of group pooled across concentrations and is scaled to the same *y*-axis as (**C**). (**D**,**E**) Individual male (**D**), M01) and female (**E**), F01) glomeruli showing sample odorant response selectivity patterns. The pseudocolored difference maps that were evoked by MV, BA, 2HEX, and 2M2B are shown (top) with all 4 corresponding fluorescent records superimposed per glomerulus (bottom). Individual traces represent 4-trial averages per odorant and the boxed portion of the traces indicates the frames that were used to generate the response maps in (**A,B** and **D,E**) and the analyses in (**C** and **F**). (**F**) The mean ± SEM number of odorants that evoked a measurable response in each glomerulus are plotted for male and female glomerular populations. *N*s indicate the number of glomeruli per group. The *y*-axis ranges from 1–4 because each individual glomerulus was categorized as responding to 1–4 odorants in the panel. (**G**,**H**) Glomerular response maps that were evoked by 15 au 2HEX in a representative male mouse (**G**), M06) and a representative female mouse (**H**, F10). (**I**) Fluorescent records that correspond to the glomerular callouts in (**G**,**H**). The boxed portion of the fluorescent records indicates the frames that were used to generate the peak response maps in (**G**,**H**) and the analyses that are summarized in (**J**). Solid lines ± shaded regions represent the mean ± SEM fluorescent record across 4 repeated trials for each glomerulus. Each trace is scaled relative to its individual maximum. (**J**) Mean ± SEM odorant-evoked change in fluorescence (ΔF) plotted as a function of odorant concentration. The inset shows the main effect of group pooled across concentrations and is scaled to the same *y*-axis as J.

As shown by the representative methyl valerate (MV)-evoked difference maps in Fig. 1A,B and the dot plot in Supplementary Fig. S1, the overall number of odorant-evoked glomerular responses observed in these unmanipulated female subjects was significantly greater than that observed in unmanipulated male subjects (Fig. 1C, inset, main effect of sex, *F*_(1,17) = _11.199, *p* = 0.004, η_p_^2^ = 0.397). Additionally, the observed difference between sexes was larger at higher odorant concentrations (Fig. 1C; sex × concentration interaction, *F*_(2,34)_ = 3.876, *p* = 0.030, η_p_^2^ = 0.186). *Post hoc* analyses evaluating the 2-way interaction confirmed that there was an effect of concentration in both sexes, as expected. However, this effect was larger in females (*F*_(2,20)_ = 21.172, *p* < 0.001, η_p_^2^ = 0.679) than in males (*F*_(2,14)_ = 10.997, *p* = 0.001, η_p_^2^ = 0.611), indicating that concentration-dependent glomerular recruitment^36^ is augmented in females.

The sex difference in the number of glomeruli receiving measurable odorant-evoked OSN input (Fig. 1A–C) could be caused by a difference in odorant response selectivity, such that the OSNs projecting to each glomerulus respond to more odorants in females than in males. To test this possibility, we assessed the odor tuning of each responsive glomerulus by quantifying the number of odorants in the panel that evoked a measurable response in that glomerulus. For example, the two sample glomeruli in Fig. 1D,E only received input from OSNs that were stimulated by a single odorant from the 4-odor panel. On average, glomeruli from females responded to slightly (8.6%) but significantly more odorants than glomeruli from males (Fig. 1F; *t*_(df = 632.9)_ = −2.855, *p* = 0.004). Even a slight shift in tuning would be sufficient to cause a robust difference in the number of odorant-evoked glomerular responses because certain glomeruli might exhibit no response to our odor panel in more narrowly tuned male OSNs, but might respond to just one of the odorants in more broadly tuned female OSNs. We employed 4 structurally- and perceptually-disparate odors in our main odorant test panel, but we note that a much larger number of odorants would need to be screened to determine if the sex-dependent tuning of OSN output might differ depending on odor identity.

An alternative explanation would be that the sex difference in the number of glomerular inputs is an artifact of our optical detection threshold, which might arise if, for instance, females exhibited larger spH responses that were easier to detect optically. To evaluate this possibility, we measured total odorant-evoked OSN synaptic output into each olfactory bulb glomerulus by subtracting 1 sec of pre-odor baseline frames from 1 sec of frames centered on the approximate peak of the spH signal (Fig. 1G–I). The average odorant-evoked ΔF was calculated across all glomeruli per odor map for each subject, and then the resulting data were analyzed via mixed-model ANOVA. On average, there was no difference between sexes in the peak odorant-evoked ΔF (Fig. 1J, inset; main effect of sex, *F*_(1,17)_ = 0.119, *p* = 0.734, η_p_^2^ = 0.007). Increasing the odorant concentration resulted in a corresponding increase in peak odorant-evoked response amplitudes (*F*_(2,34)_ = 56.891, *p* < 0.001, η_p_^2^ = 0.770), as expected. However, this concentration-dependent increase in peak odorant-evoked ΔFs was comparable between sexes (Fig. 1J, main; non-significant sex × concentration interaction, *F*_(2,34)_ = 0.965, *p* = 0.391, η_p_^2^ = 0.054). This result suggests that the noticeably different patterns of OSN synaptic input to the brain (Fig. 1A,B and G,H) are not attributable to differences in response magnitudes, and, perhaps more importantly, that the magnitude of OSN input to olfactory bulb glomeruli is similar in individual male and female subjects (Supplementary Fig. S1) despite the difference in the number of glomeruli receiving input.

### Odor-evoked OSN output is faster in females than in males

**Figure 2:**
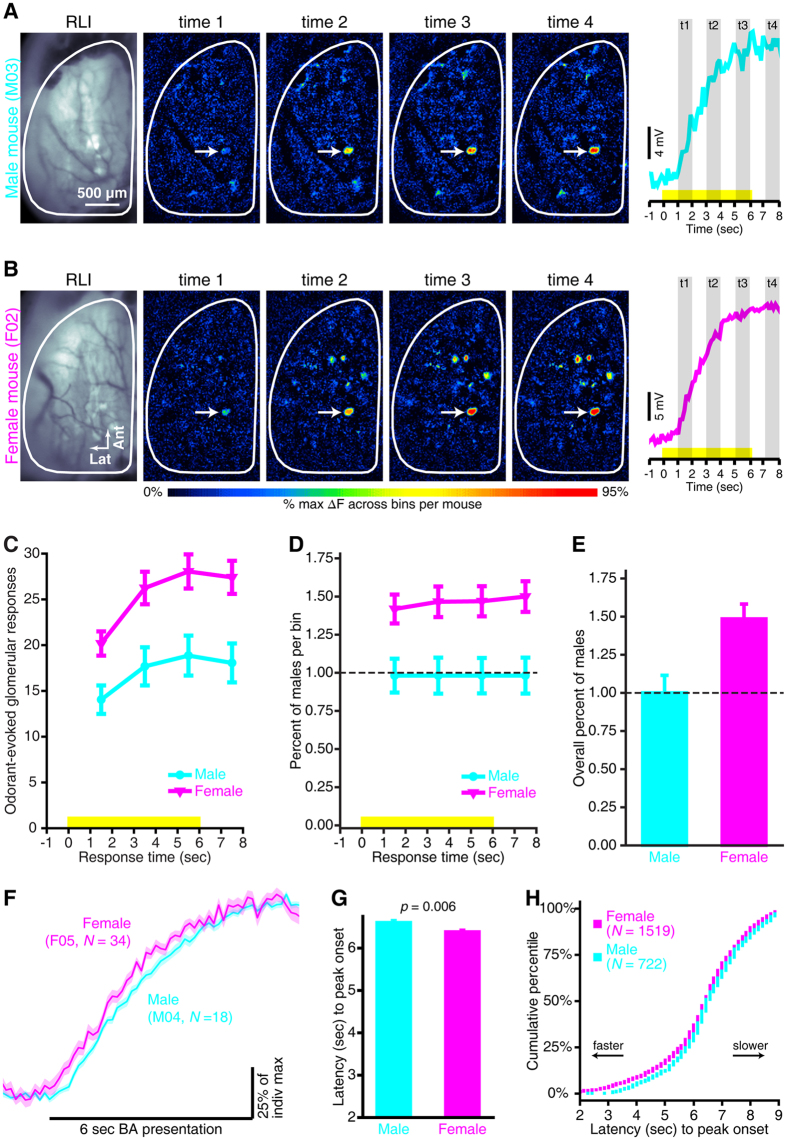
Temporal evolution of odorant-evoked OSN activity in unmanipulated females compared to unmanipulated males. (**A–E**) Sex-dependent differences in the temporal evolution of spatial odor maps. **(A,B**) RLIs and odorant-evoked difference maps that were measured during 4, 1-sec time bins from a representative male mouse (**A**), M03) and a representative female mouse (**B**), F02). Timelines illustrating the 4, 1-sec time bins (shaded regions; t1-t4, response times 1–4) relative to stimulus presentation (yellow stimulus bar) are shown to the right. Example traces are superimposed on each timeline and correspond to the glomerular callouts (white arrows) in (**A**,**B**). Each trace represents the average fluorescent record for that glomerulus across 4 trials of 15 au 2M2B. (**C**) Odorant-evoked glomerular responses plotted as a function of time relative to stimulus presentations (yellow bar). Times are plotted to correspond with the middle of each 1-sec bin. For example, data corresponding to time 1 (which was an average of 7 frames acquired during 1–2 sec after odorant onset) is plotted at 1.5 sec. These data are calculated across all concentrations (7.5 au, 15 au, and 30 au) of all odors (BA, MV, 2HEX, and 2M2B). (**D**) To show proportional differences in the number of odorant-evoked glomerular responses throughout the stimulus presentation, the data in **C** were normalized relative to the male group within each bin and are plotted as the percent of males as a function of response time. **(E)** Overall ratio of glomerular responses relative to males, pooled across all 4 time bins. Dashed lines in (**D**,**E**) indicate 100% of male activity. (**F**–**H**) Odorant-evoked spH signals reach maximum response magnitudes slightly faster in intact-females than in intact-males. (**F**) BA-evoked spH signals compared between representative male (M04, cyan) and female (F05, magenta) subjects. Solid lines ± shaded regions, mean ± SEM BA-evoked response across glomerular responses per subject; *N*s, number of BA-responsive glomeruli per subject. (**G**) Overall, when averaged across odorants and between sexes the mean ± SEM latency to peak onset is ~0.31 sec faster in intact-females than in intact-males. (**H**) Cumulative probability plot showing the male and female distributions of peak latency values pooled across individual fluorescent records.

Odor information is not only encoded by the static pattern of activity that is mapped across the glomerular layer of the bulb, but also by the temporal dynamics of that activity^36^,^37^,^38^. To test whether the degree of the female-specific enhancement (Fig. 1A–C) varies across the duration of the odorant presentation we separated the spH signals into 4 1-sec time bins (Fig. 2A,B), and then analyzed the data via a mixed, 4-way ANOVA and additional planned *post hoc* tests. There were a larger number of glomeruli receiving OSN input in females, not only at the peak of the spH response (Fig. 1A–C and time 4 in Fig. 2A,B), but also during all earlier response times (Fig. 2A–C; time 1, *F*_(1,17)_ = 8.985, *p* = 0.008, η_p_^2^ = 0.346; time 2, *F*_(1,17)_ = 9.763, *p* = 0.006, η_p_^2^ = 0.365; time 3, *F*_(1,17)_ = 10.242, *p* = 0.005, η_p_^2^ = 0.376).

As illustrated by Fig. 2A–C, the number of glomeruli receiving measurable OSN input increases with the time elapsed since odorant onset (main effect of time bin, *F*_(3,51)_ = 110.030, *p* < 0.001, η_p_^2^ = 0.866). This likely reflects the gradual increase in spH fluorescence as OSNs release neurotransmitter over time, which rapidly exceeds our optical detection threshold for strongly activated glomeruli and more slowly exceeds our optical detection threshold for very weakly activated glomeruli. However, there was also a significant sex × time bin interaction (Fig. 2C; *F*_(3,51)_ = 7.428, *p* < 0.001, η_p_^2^ = 0.304), showing that the number of responsive glomeruli increased faster in females than in males (which cannot be the result of an optical detection threshold). To test whether the increased number of responsive glomeruli in females developed over time, we calculated the ratio of odorant-evoked glomerular responses in females relative to that in males for each response time bin. This qualitative analysis shows that even though the number of glomeruli receiving measurable OSN input increased throughout the odor presentation, this number was proportionately larger for females than males during all 4 measured time points (Fig. 2D), averaging 48% ± 0.1% more responsive glomeruli overall (Fig. 2E).

Even though the magnitude of the peak spH responses was the same between sexes (Fig. 1J), the time course-dependent sex differences in static spatial maps (Fig. 2A–E) suggested that there might be subtle differences in the temporal dynamics of those signals. There is precedent in this, where odorant-evoked spH signals in OMP knockout mice eventually reach comparable peak magnitudes to that in OMP-expressing mice^39^, despite having relatively slowed OSN response kinetics and decreased sensitivity^40^. Careful inspection of the fluorescence records in Fig. 1I suggests that similar differences in response time course might exist between sexes because the peak amplitudes (boxed portion of traces) are comparable, but the slopes of the signals appear to differ during earlier (pre-peak) response times. While spH does not clearly illustrate the dynamics of individual inhalations, it does usefully report on the cumulative total odorant-evoked OSN output within each glomerulus over the course of an odorant presentation. To further evaluate potential sex differences in the time course of odorant-evoked nerve output, particularly during the relatively early, pre-peak part of the responses, fluorescence traces from all glomerular ROIs were normalized to their individual minima and maxima. To visualize the timing of odorant-evoked spH signals, the normalized traces were separately pooled across glomeruli per odorant within a mouse, and the average odorant-evoked spH waveforms that were evoked by a given odorant in individual mice were plotted together for qualitative evaluation (as in Fig. 2F).

Odorant-evoked OSN output appeared to increase slightly faster in females than in males (Fig. 2F), reaching the plateau of the response slightly earlier. We quantified the latency (in sec) to onset of the peak response plateaus (which are relatively prolonged; e.g., Figs 1I and 2F) and looked for group differences between individual animals (Fig. 2G; *N*_Male_ = 8, *N*_Female_ = 11) as well as between distributions of latency values pooled across glomerular fluorescence records from all animals (Fig. 2H). On average, the onset of peak odorant-evoked response magnitudes occurred ~0.31 sec earlier in females than in males (Fig. 2G; *F*_(1,17)_ = 9.861, *p* = 0.006, η_p_^2^ = 0.367). This subtle difference in timing exceeds behaviorally-relevant timescales because sensory processing that is sufficient to support odor detection and discrimination occurs in under 200 ms^41^,^42^,^43^.

### Higher contrast between odorants in the primary sensory odor representations of females than males

What could be the functional significance of the spatiotemporal sex differences in odorant-evoked OSN activity that were observed here (Figs 1 and 2)? Fundamentally, the spatial pattern of OSN input across glomeruli is the initial representation of odor identity in the brain. The degree of difference between these spatial patterns of glomerular activity predicts the perceptual differences between odors^44^,^45^, so we quantified the differences between the representations of different odor pairs within male and female mice.

**Figure 3:**
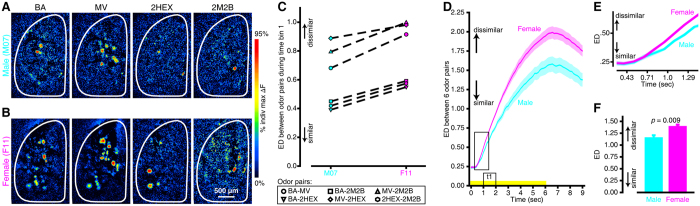
Sex differences in the contrast between the primary sensory representations of different odors. (**A,B**) BA-, MV-, 2HEX-, and 2M2B-evoked difference maps from representative male (**A**), M07) and female (**B**), F11) mice. (**C**) Euclidean distance (ED) between 6 pairwise odor map comparisons for the static maps that are shown in (**A** and **B**). (**D**) Mean ± SEM ED between all 6 odor pairs from all male (cyan) and female (magenta) subjects across 64 frames that correspond to 0–9 sec relative to odorant onset. The yellow stimulus bar indicates the time of odorant presentations and the boxed region of the stimulus bar (t1, time bin 1) notes the frames that were used to generate the difference maps in (**A,B**) and the corresponding ED comparisons shown in **C**. (**E**) Enlargement of the boxed region of the frame-by-frame ED analysis in (**D**) shows that odor maps are more dissimilar in females than in males by as early as 1 sec into the odorant presentations. (**F**) Overall mean ± SEM ED between odor representations pooled across 6 odor pairs and 64 frames.

Differences between odorant-evoked glomerular response maps were quantified as Euclidean distances (EDs) in *N*-dimensional vector space, where *N* was equivalent to the number of glomerular ROIs identified in each mouse. Smaller EDs indicate that neural representations of stimuli within a pair are more similar to each other, whereas larger EDs indicate that the stimulus representations are more dissimilar. We calculated the pairwise ED for all 4 odors in our panel, yielding 6 possible odor pairs: BA-MV, BA-2HEX, BA-2M2B, MV-2HEX, MV-2M2B, and 2HEX-2M2B. Figure 3A,B shows BA-, MV-, 2HEX-, and 2M2B-evoked response maps that were generated for time bin 1 (the first rising phase of the odor response – see Fig. 2A,B) from representative male and female subjects. Pairwise comparisons across odor maps within each subject show that all pairs are discriminable by this time point (Fig. 3C; EDs greater than 0), but that the maps are more easily distinguished in the female mouse than in the male mouse (Fig. 3C; F11 has larger EDs for all 6 odor pairs). We analyzed the 6 pairwise EDs for all subjects during time bin 1 with a sex × odor pair ANOVA and confirmed that, on average, odor maps were more dissimilar in females than in males during this relatively early response time bin (mean ± SEM ED between odor maps; female group = 0.707 ± 0.015, male group = 0.637 ± 0.019, main effect of sex *F*_(1,16)_ = 7.942, *p* = 0.012, η_p_^2^ = 0.332).

Because individual maps for a given odorant evolve over time throughout the duration of a stimulus presentation (e.g., Fig. 2A,B), we extended this analysis and calculated EDs between odor pairs over 64 consecutive frames that began at time = 0 sec relative to stimulus onset (Fig. 3D). While the ED between odor pairs got larger over time for all subjects (main effect of frame number; *F*_(63,1008)_ = 389.539, *p* < 0.001, η_p_^2^ = 0.961), overall the odor representations were more dissimilar in females than they were in males (Fig. 3F; group means ± SEMs pooled across 64 frames, *F*_(1,16)_ = 8.844, *p* = 0.009, η_p_^2^ = 0.356). Additionally, the enhanced contrast between sensory maps that was observed in females occurred relatively early in the trial and became larger over time (Fig. 3D; sex × frame interaction, *F*_(63,1008)_ = 7.200, *p* < 0.001, η_p_^2^ = 0.310). In fact, this frame-by-frame analysis reveals that the sex difference was detectable in these data as early as ~0.75 sec after odorant onset (Fig. 3E). The more numerous, faster OSN outputs in females thus enhance the differences between odor representations.

### The number of glomeruli receiving odorant-evoked OSN input is influenced by circulating gonadal hormones in males and females

The sexually dimorphic odor-evoked activation of olfactory bulb glomeruli that we observed in unmanipulated males and females could be the result of organizational differences that are differentiated early in life, or it could alternatively be attributed to activational effects of circulating sex hormones on the peripheral olfactory system. To test these possibilities, we performed the same *in vivo* optical imaging procedures on an additional 43 mice 2 weeks after those subjects underwent bilateral gonadectomy or sham-control surgical procedures (Supplementary Fig. S2). If the observed sex differences (Figs 1, 2 and 3) are attributable to structural differences in the organization of this olfactory pathway, then the removal of circulating sex hormones through gonadectomy should have no effect on odorant-evoked glomerular response maps. Conversely, if odorant-evoked OSN neurotransmitter release is susceptible to hormonal modulation, then gonadectomy should induce changes in odor-evoked OSN activity between sham-manipulated and gonadectomized mice of each sex. If circulating sex hormones are the *only* cause of the difference between male and female OSN responses, then gonadectomy should make the response maps in male and female mice equivalent.

**Figure 4:**
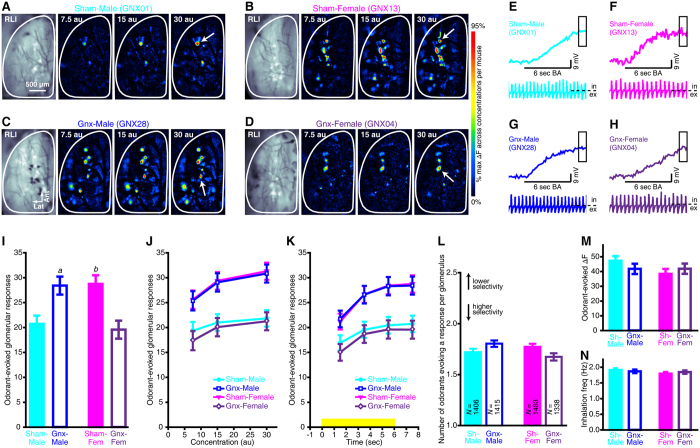
Sexually dimorphic activation of olfactory bulb glomeruli is dependent upon circulating gonadal hormones. (**A–D**) RLI images through the cranial window and pseudocolored difference maps showing the peak responses evoked by 3 concentrations of BA from a Sham-male **(A,** GNX01), a Sham-female (**B**), GNX13), a Gnx-male (**C**), GNX28), and a Gnx-female (**D**), GNX04). **(E**–**H**) Fluorescent records from individual trials that correspond to the glomerular callouts that are noted by white arrows on the 30 au BA-evoked maps in (**A**–**D**). The boxed portion of the fluorescent records indicates the frames that were used to generate response maps in (**A**–**D**) and analyses summarized in (**I**,**J**,**L** and **M**). The example piezosensor recordings that are shown below each response amplitude are from a single trial of 30 au BA. Positive and negative portions of each respiration trace respectively correspond to inhalation (in) and exhalation (ex) phases of the respiratory cycle. (**I**) Odorant-evoked glomerular responses during the peak response phase are pooled across all odorants and concentrations and plotted separately for each group. *a* indicates *p* = 0.008 when compared with Sham-male and Gnx-female groups; *b* indicates *p* = 0.001 when compared with Sham-male and Gnx-female groups. (**J**) Odorant-evoked glomerular responses are pooled across odorants and plotted as a function of concentration for each group. (**K**) Odorant-evoked glomerular responses are plotted as a function of time relative to stimulus presentation (yellow stimulus bar). (**L**) The number of odorants that evoked a measurable response in each glomerulus are plotted for sex × surgical treatment glomerular populations. *N*s indicate the number of glomeruli per group. Note that each individual glomerulus was categorized as responding to 1, 2, 3, or 4 odorants in the panel, but the *y*-axis is truncated at 2.5 to display an appropriate range for the group means. (**M**) Peak odorant-evoked change in fluorescence (ΔF) pooled across all odorants and concentrations for each group. (**N**) Inhalation frequency during 6 sec odorant presentations for each group. Data are pooled across respiration measurements that were recorded during trials from each concentration of each odorant. The data shown in (**I**–**N**) are plotted as the mean ± SEM.

The number of glomeruli receiving measurable OSN input was quantified for each odorant and concentration per mouse and analyzed via a mixed, 4-way ANOVA and additional *post-hoc* tests. Comparison of Sham-manipulated females and Sham-manipulated males replicated our initial findings (Fig. 1A–C), revealing a greater number of glomeruli receiving odorant-evoked nerve input in Sham-females than in Sham-males (compare Fig. 4A and B; *F*_(1,21)_ = 16.104, *p* < 0.001, η_p_^2^ = 0.434). Critically, we also observed an interaction between sex and surgical treatment (Fig. 4I, *F*_(1,39)_ = 23.250, *p* < 0.001, η_p_^2^ = 0.373; and see Supplementary Fig. S3). Regardless of the odorant being presented (Supplementary Fig. S4), in females the removal of the ovaries resulted in a significant reduction in the number of glomeruli receiving measurable odorant-evoked OSN input (Fig. 4I and also compare Fig. 4B and D; *F*_(1,19)_ = 15.577, *p* < 0.001, η_p_^2^ = 0.451), causing the Gnx-females to have odor maps that were similar to those observed in the Sham-manipulated males (Fig. 4I and also compare Fig. 4A and D; non-significant difference in odorant-evoked glomerular responses, *F*_(1,20)_ = 0.222, *p* = 0.642, η_p_^2^ = 0.011). Interestingly, orchiectomy had the opposite effect on males, such that Gnx-males had a greater number of odorant-evoked glomerular responses than Sham-manipulated males (Fig. 4I and also compare Fig. 4A and C; *F*_(1,20)_ = 8.744, *p* = 0.008, η_p_^2^ = 0.304), and were thus more similar to Sham-females (Fig. 4I and also compare Fig. 4B and C; non-significant difference in odorant-evoked glomerular responses, *F*_(1,19)_ = 0.019, *p* = 0.892, η_p_^2^ = 0.001).

When we pooled across odorants and looked at odorant-evoked glomerular response maps over a range of concentrations we found that higher odorant concentrations tended to recruit more glomeruli than lower concentrations (effect of concentration, *F*_(2,78)_ = 75.476, *p* < 0.001, η_p_^2^ = 0.659). However, we identified a sex × group × concentration interaction (Fig. 4A–D and J; *F*_(2,78)_ = 5.655, *p* = 0.005, η_p_^2^ = 0.127) that suggested concentration-dependent glomerular recruitment was not equivalent among sex × surgical treatment groups. The largest effects of increasing concentration on glomerular recruitment were observed in Sham-female and Gnx-male groups (which were equivalent to each other; non-significant 2-way interaction, *F*_(2,38)_ = 0.040, *p* = 0.961, η_p_^2^ = 0.002), whereas concentration-dependent glomerular recruitment was relatively less-robust in both Sham-male and Gnx-female groups (which were equivalent to each other; non-significant 2-way interaction, *F*_(2,40)_ = 0.760, *p* = 0.474, η_p_^2^ = 0.037). Gonadectomy thus appears to attenuate concentration-dependent glomerular recruitment in females, but enhance it in males (Fig. 4J).

We next separated the spH signals into 4 1-sec time bins (Supplementary Fig. S5) and analyzed the data via a 5-way ANOVA, which yielded sex × surgical treatment (*F*_(1,39)_ = 19.058, *p* < 0.001, η_p_^2^ = 0.328) and sex × surgical treatment × time bin interactions (Fig. 4K; *F*_(3,117)_ = 40.888, *p* < 0.001, η_p_^2^ = 0.512). After performing additional *post-hoc* factorials, we once again confirmed that there were a larger number of odorant-evoked glomerular responses in intact females relative to intact males not only at the peak of spH response (Fig. 4I and K, time = 7–8 sec), but also during all earlier response times (Fig. 4K; time = 1–2 sec, *F*_(1,21)_ = 5.614, *p* = 0.027, η_p_^2^ = 0.211; time = 3–4 sec, *F*_(1,21)_ = 14.178, *p* < 0.001, η_p_^2^ = 0.403; time = 5–6 sec, *F*_(1,21)_ = 15.837, *p* < 0.001, η_p_^2^ = 0.376). Notably, this difference was reversed by gonadectomy (Supplementary Fig. S5) because the number of odorant-evoked glomerular responses was proportionately reduced in Gnx-females relative to Sham-manipulated females throughout the duration of the trial (Fig. 4K; time = 1–2 sec, *F*_(1,19)_ = 8.724, *p* = 0.008, η_p_^2^ = 0.315; time = 3–4 sec, *F*_(1,19)_ = 12.037, *p* = 0.003, η_p_^2^ = 0.388; time = 5–6 sec, *F*_(1,19)_ = 13.685, *p* = 0.002, η_p_^2^ = 0.419), whereas a proportional enhancement was observed throughout the trial in Gnx-males relative to Sham-manipulated males (Fig. 4K; time = 1–2 sec, *F*_(1,20)_ = 3.818, *p* = 0.065, η_p_^2^ = 0.160; time = 3–4 sec, *F*_(1,20)_ = 7.791, *p* = 0.011, η_p_^2^ = 0.280; time = 5–6 sec, *F*_(1,20)_ = 9.001, *p* = 0.007, η_p_^2^ = 0.310).

Consistent with findings from the first experiment (Fig. 1F), the differences between groups in the spatial arrangement of glomerular odor representations may be related to slight changes in odor tuning of individual glomeruli. We observed a small but significant interaction of sex and surgical treatment on the number of odorants that evoked a response in each glomerulus (Fig. 4L; *F*_(1,2817)_ = 7.644, *p* = 0.006, η_p_^2^ = 0.043). The interaction suggested that glomerular selectivity was increased by gonadectomy in females since glomeruli from Gnx-females tended to respond to slightly fewer odorants than Sham-females (Fig. 4L). By contrast, glomeruli from Gnx-males responded to slightly more odorants than from Sham-males (Fig. 4L), suggesting that gonadectomy in males may have broadened the tuning of individual glomeruli.

Similar to the first experiment (Fig. 1G–J), there were no overall differences between groups in peak response magnitudes (Fig. 4E–H, boxed portion of traces; Fig. 4M, non-significant sex × surgical treatment interaction, *F*_(1,39)_ = 1.768, *p* = 0.191, η_p_^2^ = 0.043; Supplementary Fig. S3). Additionally, the effects reported here cannot be attributed to differences in respiration because they were identified in anesthetized imaging preparations in which depth of anesthesia and respiration frequency were constant throughout the duration of the experiment and did not differ between any groups (see sample respiration traces in Fig. 4E–H and group means in Fig. 4N; non-significant sex × surgical treatment interaction, *F*_(1,37)_ = 0.802, *p* = 0.376, η_p_^2^ = 0.021).

These data demonstrate that OSN physiology may normally be modulated by circulating sex hormones in both female and male mice. It is possible that this modulation could fluctuate in females in correlation with the fluctuating levels of sex hormones that are associated with different phases of the estrous cycle. Although Sham-manipulated females exhibited normal 4 day estrous cycles (Supplementary Fig. S2), we saw no obvious relationship between estrous cycle phase and the number of odorant-evoked glomerular responses (Supplementary Fig. S3). However, we note that the small number of animals in each phase of the cycle does not permit definitive conclusions.

### Gonadal hormones modulate the timing and discriminability of odorant-evoked OSN activity

To assess the potential influence of circulating gonadal hormones on OSN response timing and the contrast between odorant-evoked glomerular response maps, we repeated the analyses shown in Figs 2 and 3 with the data from sham-manipulated and gonadectomized mice.

**Figure 5:**
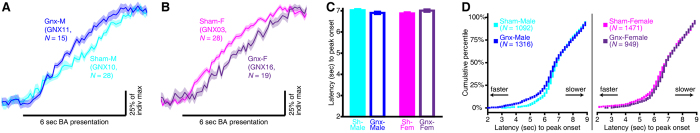
Odorant-evoked spH signals are accelerated by gonadectomy in males, but slowed by gonadectomy in females. (**A,B**) BA-evoked spH signals compared between representative Sham-male (GNX11) and Gnx-male (GNX10) subjects (**A**) and also between representative Sham-female (GNX03) and Gnx-female (GNX16) subjects (**B**). The solid lines ± shaded regions represent the mean ± SEM fluorescent record across all BA-evoked glomerular responses per subject, and the *N*s indicate the number of glomeruli that are contributing to each subject’s mean BA-evoked spH signal. **(C)** Mean ± SEM latency to onset of peak spH signal from sex × surgical treatment groups. (**D**) Cumulative probability plots showing the distributions of peak latency values pooled across individual fluorescent records. The *N*s indicate the number of odorant-evoked spH signals pooled across odorants.

The timing of odorant-evoked OSN neurotransmitter release was compared between Sham-male and Gnx-male subjects (Fig. 5A) as well as between Sham-female and Gnx-female subjects (Fig. 5B) (also see Fig. 4E–H and compare slopes of traces). Intriguingly, the removal of circulating gonadal hormones resulted in opposite effects on temporal properties of OSN physiology in males and females (Fig. 5C; sex × surgical treatment interaction, *F*_(1,39)_ = 6.092, *p* = 0.018, η_p_^2^ = 0.135). Regardless of which odorant was presented (non-significant odorant × sex × surgical treatment interaction, *F*_(3,117)_ = 1.447, *p* = 0.233, η_p_^2^ = 0.036), the onset of peak responses in Gnx-females was ~0.18 sec delayed relative Sham-females, whereas the onset of peak responses in Gnx-males was ~0.18 sec earlier than that in Sham-males. Though individual latency values from Gnx-females tended to be slightly larger (i.e., slower responses) than those from Sham-females (Fig. 5D, right panel; K-S, *Z* = 1.428, *p* = 0.034), they were equivalent to latency values in Sham-males (K-S, *Z* = 0.960, *p* = 0.315), which were also larger than those in Sham-females (K-S, *Z* = 2.125, *p* < 0.001). Conversely, individual latency values from Gnx-males were smaller (i.e., faster responses) than those from Sham-males (Fig. 5D, left panel; K-S, *Z* = 1.992, *p* < 0.001), but equivalent to those from Sham-females (K-S, *Z* = 0.565, *p* = 0.906). Importantly, these effects were not attributable to differences in respiration (Fig. 4N).

**Figure 6:**
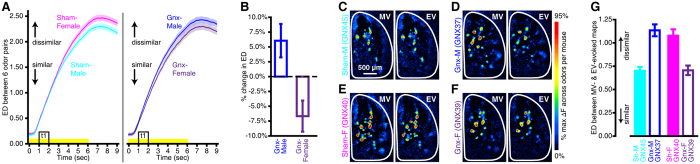
Odor maps become relatively more discriminable after gonadectomy in males, but relatively less discriminable after gonadectomy in females. (**A**) Odor pairs tended to be further apart in Euclidean space (i.e., more dissimilar) in Sham-females than in Sham-males (left panel), and this difference was reversed by gonadectomy (right panel). The mean ± SEM ED between all 6 odor pairs is plotted across 64 frames that correspond to 0–9 sec relative to odorant onset. The yellow stimulus bar indicates the time of odorant presentations and the boxed region of the stimulus bar (t1, time bin 1) notes the frames that were used to generate the difference maps in (**C**–**F**). (**B**) The effects of gonadectomy on primary sensory odor representations are plotted as the percent change in ED relative to Sham-control groups. The dashed line indicates no change relative to Sham-controls. Values above and below the dashed line respectively note relative increases and decreases in odor map discriminability. (**C**–**F**) Pairs of MV- vs EV-evoked difference maps from a representative Sham-male (C, GNX45), Gnx-male (**D**), GNX37), Sham-female (**E**), GNX40), and Gnx-female (**F**), GNX39). (**G**) Mean ± SEM ED between MV- and EV-evoked maps across all 64 frames for each subject shown in (**C**–**F**).

When we quantified the ED between pairs of odor maps as above, we found that odor pairs tended to be further apart in Euclidean space (i.e, more dissimilar) in Sham-manipulated females than in Sham-manipulated males (Fig. 6A, left panel), replicating our previous results (Fig. 3). However, the reverse pattern was observed between Gnx-females and Gnx-males (Fig. 6A, right panel), which suggests that while coarse odor discrimination between glomerular activity maps that are evoked by different odorants may be better in the gonad-intact female than in the gonad-intact male (e.g., Fig. 3A–C), coarse discrimination between glomerular odor maps may be poorer in Gnx-females than in Gnx-males. The interactions between sex and surgical treatment (*F*_(1,39)_ = 5.891, *p* = 0.020 η_p_^2^ = 0.131) and between sex, surgical treatment, and frame number (*F*_(63,2457)_ = 3.689, *p* < 0.001 η_p_^2^ = 0.086) suggested that this was because odor pairs tended to be slightly more similar to each other in Gnx-females than they were in Sham-females (Supplementary Fig. S6), whereas those same odor pairs tended to be slightly more dissimilar from each other in Gnx-males than they were in Sham-males (Supplementary Fig. S6). To illustrate this point, we divided the overall ED between all 6 odor pairs across all 64 frames from each Gnx group by the overall ED from their corresponding Sham-control group (Fig. 6B). These ratios were plotted as the percent change in ED between odor pairs relative to that in Sham-controls, and they show that gonadectomy resulted in increased odor map discriminability in males and decreased odor map discriminability in females (Fig. 6B).

In a subset of subjects the odor panel was expanded to include the odorant ethyl valerate (EV) to provide a close comparison to the odorant methyl valerate (MV) during *in vivo* optical imaging. In these subjects there was a significant interaction in the effects of sex and surgical treatment on the discriminability between primary sensory representations of MV and EV (Fig. 6C–G; *F*_(1,14)_ = 6.784, *p* = 0.021, η_p_^2^ = 0.326), as well as on the number of glomerular responses that were evoked by these 2 chemically-similar odorants (Supplementary Fig. S7). Specifically, MV- and EV-evoked glomerular response maps tended to be more similar to each other (i.e., harder to discriminate) in Sham-males than they were in Sham-females (Fig. 6G). By contrast, MV- and EV-evoked glomerular response maps tended to be more dissimilar from each other (i.e., easier to discriminate) in Gnx-males than they were in Gnx-females (Fig. 6G). This result demonstrates that neuroendocrine factors can influence the representation of odor identity at the input to the brain, such that circulating gonadal hormones likely help females in making challenging olfactory discriminations but may make such discriminations more difficult for males.

## Discussion

In the present experiments we observed a sex difference in odorant-evoked signaling from the OSNs in the olfactory epithelium to the brain’s olfactory bulb. Odorant presentation in unmanipulated female mice elicited OSN input into a broader range of olfactory bulb glomeruli than in males. Though responsive glomeruli received the same magnitude of OSN input on average between males and females, in females this input occurred earlier in the odor presentation. As a result, different odorants evoked more different spatial patterns of OSN input to the brain in females than males, even within the first second of odor presentation. Gonadectomy experiments revealed that circulating sex hormones may influence these responses. Gonadectomized females exhibited slower OSN responses in fewer glomeruli than control females, while gonadectomized males exhibited faster OSN responses in more glomeruli than control males. These results suggest that gonadal hormones may facilitate odor detection and discrimination of similar odorants in females but impair it in males.

Gonadectomy did not eliminate the sex difference between males and females but in fact reversed it, suggesting that the functional neuronal circuits underlying the sex-specific peripheral olfactory input to the brain exist in both males and females^46^. Sexually dimorphic activation of sensory pathways presumably underlies many of the obvious differences in male and female behavior^28^,^47^, and if both sexes are developmentally programmed with the same underlying circuitry then they may also be capable of displaying the same sex-specific behaviors in response to sensory stimuli^46^. The reversal of sex-specific olfactory coding after gonadectomy implies a role for hormones in dynamically shaping the activity of sexually dimorphic sensory circuitry in the olfactory bulb. The functioning of these olfactory pathways could be further modulated by experiential factors^2^,^48^ since the within-cage olfactory environments of same-sex-housed males and females are different, as are the cage environments of mice with different endocrine statuses^28^,^47^ (i.e., sham-operated versus gonadectomized).

It is suggestive that these results parallel the common observation that females exhibit superior olfactory capabilities than males^1^,^4^,^9^,^10^,^11^,^12^,^13^,^14^. Sensory performance in a noisy environment is typically modeled as information accumulation over time, as demonstrated by the speed-accuracy tradeoff for odor detection and discrimination^42^,^49^. The larger number of glomeruli receiving input in females (Fig. 1) means that females receive more total olfactory sensory input to the brain, while the more rapid OSN response (Fig. 2) gets that information to the brain earlier in females than in males. How would the larger number of glomerular responses in females affect odor discrimination performance? In principle the modest broadening of glomerular odor tuning could impair discrimination by increasing overlap in response maps, but conversely the greatly increased number of responding glomeruli in females provides a much richer set of inputs for the brain to interpret. The utility of the Euclidean distance metrics reported here is that they quantify the actual difference in neural odorant representations, taking both the number of glomeruli and overlap of responses into account, and the larger EDs in females demonstrates that the neural response patterns are more different across odorants in females than they are in males. The finding that ovariectomy reduces these computational advantages in females is consistent with previous reports that estrogen replacement therapy protects against olfactory impairment in post-menopausal women^22^,^23^ and also in ovariectomized rats with lesions to the olfactory epithelium^25^.

What is the mechanism of the difference in OSN coding between males and females? Male and female mice have the same number of OSNs in the olfactory epithelium^50^, so the difference presumably arises physiologically, potentially from differences in the odor selectivity of OSNs (Fig. 1F) or differences in OSN response kinetics (Fig. 2F,H). The results from the gonadectomy-imaging experiment suggest that circulating gonadal hormones could influence those aspects of OSN odor processing, albeit in different directions in males and females. Notably, in these mice spH is expressed under the OMP promoter and the odor tuning, temporal properties, and sensitivity of OSNs are known to be influenced by OMP expression^39^,^40^,^51^. OMP expression might be susceptible to hormonal modulation^52^ that could conceivably result in sex-specific activation of OSNs. Regardless of potential interactions with OMP, a neuroendocrine mechanism could only be confirmed through the restoration of individual hormones such as estradiol or dihydrotestosterone. Without performing such a hormone replacement experiment we do not know for certain if gonadal hormones cause the differences in olfactory sensory processing that were observed here, nor can we rule out the possibility of contributions from other endocrine effects because the gonadectomy surgical procedure can also result in several side effects, such as increased secretion of gonadotropins and alterations to the hypothalamic-pituitary system^53^,^54^. Nonetheless, the locus of these putative neuroendocrine interactions could be peripheral. Higher levels of odorant-binding protein genes have been observed in olfactory epithelia from female mice than from male mice^55^, which is consistent with the decreased OSN response latencies that we observed in unmanipulated females relative to males and suggests that gonad-intact females may have a more efficient odorant-transport system. Such differences may be dynamically regulated by varying levels of sex steroids that are synthesized locally in the epithelium^56^ or that are present in other structures in the mucosa. For example, in male mice, relatively high levels of testosterone have been found in the lateral nasal gland^57^, a structure that secretes odorant-binding proteins into the epithelium. There could also be an indirect effect of gonadal hormones on olfactory transduction via modifications to the anatomy of the olfactory mucosa^20^,^22^.

Alternatively, the observed effects of gonadal hormones on OSNs could be mediated in the glomerular layer of the olfactory bulb, where populations of periglomerular (PG) interneurons and short axon cells (SACs) influence primary sensory odor coding. PG interneurons directly mediate local gain control on OSN output via GABA-mediated presynaptic inhibition of OSN synaptic terminals^58^. The activity of this intraglomerular inhibitory circuitry is in turn shaped by extensive interglomerular connections that arise from SACs, which co-express GABA and TH^59^,^60^, and GABAergic^61^,^62^,^63^,^64^,^65^ and dopaminergic^66^,^67^ signaling in the brain are subject to modulation by gonadal steroids.

Neuroendocrine effects in the glomerular layer could, for example, be mediated through estrogen receptors^68^,^69^. Aromatase, which is the enzyme necessary for the conversion of androgens to estrogens, is also expressed throughout the olfactory bulb^70^,^71^, indicating that synthesis of estradiol occurs locally in the bulb. Notably, aromatase is broadly expressed in SACs in the glomerular layer^71^, suggesting that the discriminability of different odor maps may indeed be related to estrogen-dependent regulation of interglomerular processing. Consistent with this, male olfactory bulbs contain a greater number of TH-positive neurons than female olfactory bulbs in both rodents^72^ and humans^73^. Additionally, TH mRNA in the olfactory bulb is increased by ovariectomy in female mice, and this effect can be reversed when ovariectomized mice are treated with estradiol^66^. We thus speculate that gonadal hormones may play a role in tuning olfactory bulb circuitry by modulating SACs, which could potentially include direct effects on OSN synaptic terminals via dopamine receptors^74^ as well as indirect effects on OSN signaling via connections with PG cells. It might also be possible for neuroendocrine effects to directly influence PG cell-mediated GABAergic presynaptic inhibition of OSNs. Gonadectomy in females results in an increase in GABA content in the rat brain^63^, whereas GABA content is decreased by gonadectomy in males^61^. This seems consistent with a system that exhibits relatively decreased presynaptic inhibition in gonad-intact females relative to gonad-intact males.

In rodents, there are notable sex differences in the accessory olfactory system that are essential for processing pheromone cues that are used to guide normal social and reproductive behaviors^47^,^75^. It is increasingly understood that the main olfactory system is also involved in processing biologically relevant odors^76^,^77^,^78^, though few studies have examined sex differences in processing such stimulie.g., refs 79 and 80. The present work demonstrates that OSN responses to odorants can differ between males and females even in the main olfactory bulb and even for non-social odorants, which could influence the interpretation of results from olfactory research and potentially the design of flavors and fragrances.

Regardless of the exact mechanisms by which sexually dimorphic OSN signaling emerges, these data are the first to demonstrate *in vivo* sex differences in primary sensory odor coding. These results further demonstrate that OSN function may normally be influenced by circulating gonadal hormones in both sexes, suggesting that sex hormones might underlie some of the differences in how males and females perceive their olfactory environments^28^,^34^.

## Materials and Methods

### Subjects

The present experiments used a total of 62 mice that express the synaptopHluorin (spH) exocytosis indicator under the control of the olfactory marker protein (OMP) promoter^35^,^39^,^81^. The data that are summarized by Figs 1, 2, 3 came from 8 unmanipulated (gonad-intact) males and 11 unmanipulated (gonad-intact) females. The data that are shown in Figs 4, 5, 6 came from an additional 22 males and 21 females that underwent gonadectomy (or sham-control) surgical procedures 2 weeks prior to *in vivo* optical imaging (Supplementary Fig. S2). All subjects were sexually-naïve adults (8–11 months old) that were still of reproductive age. Normal estrous cycles were observed in the sham-operated females (Supplementary Fig. S2). All experiments were performed in accordance with protocols approved by the Rutgers University Institutional Animal Care and Use Committee.

### Gonadectomy surgical procedures

The data shown in Figs 4, 5, 6 came from 43 subjects that underwent either bilateral gonadectomy (Gnx) procedures (Gnx-male, *N* = 10; Gnx-female, *N* = 10) or sham-control (Sham) surgical procedures (Sham-male, *N* = 12; Sham-female, *N* = 11) 2 weeks prior to *in vivo* epifluorescence imaging (Supplementary Fig. S2).

For orchiectomies, a suprapubic midline incision was made along the lower abdomen, the skin was retracted, the linea alba was incised, and the testis, epididymis, and surrounding fat pad was exteriorized. The vas deferens and blood vessels were ligated, the testis and epididymis were excised, the remaining content was replaced through the abdominal incision, and then the process was repeated for the second gonad. For ovariectomies, a midline dorsal incision was made on the back directly below the rib cage, the skin was retracted, and the peritoneal cavity was accessed through a small incision in the muscle layer. The cranial portion of the uterine horn and vessels was ligated, the ovary and oviduct were severed, the uterus was gently replaced into the abdominal cavity, and the process was then repeated on the contralateral side. The same procedures were carried out for Sham surgeries, except that the gonads were exteriorized and then placed back into the abdominal cavity. After Gnx or Sham surgeries all subjects were singly-housed for the duration of the study. To confirm the efficacy of ovariectomy in GNX females, all subjects underwent daily cytological procedures^82^ that began 1 week after Sham or Gnx surgeries (Supplementary Fig. S2). Note that males underwent a daily “sham-smear” procedure to maintain equal treatment across all groups.

### *In vivo* optical neurophysiology recordings

Cranial windows were surgically implanted^81^,^83^,^84^, and can be seen in the examples shown in Figs 1A,B, 2A,B and 4A–D. Odorant-evoked spH signals were visualized in freely-breathing, anesthetized subjects^81^,^85^,^86^. *In vivo* optical signals were recorded on an Olympus BX51 microscope with a filter set containing HQ480/40 excitation, Q505LP dichroic, and HQ535/50 emission filters and illumination provided by a 470 nm LED. Optical signals were acquired via a back-illuminated CCD camera (NeuroCCD, SM-256, RedShirtImaging) at a pixel resolution and frame rate of 256 × 256 and 7 Hz, respectively. Respiration was monitored in a subset of subjects (*N* = 41) by a force-transducing piezosensor strip positioned just below the diaphragm^27^.

Vapor dilution olfactometry was used to present subjects with a panel of 4 monomolecular odorants that included *n*-butyl acetate (BA), methyl valerate (MV), 2-hexanone (2HEX), and *trans*-2-methyl 2-butenal (2M2B). Odorant concentrations were standardized prior to all imaging sessions via a photoionization detector (PID; ppbRAE300, RAE Systems). Both the liquid dilution of odorant in mineral oil and the dilution of the nitrogen carrier were increased or decreased as needed to achieve 3 target concentrations on the PID for each odorant. These measurements are reported here in arbitrary units (au), because they have relative validity within odorants but uncertain absolute molar concentration. For example, the three reported concentrations of MV, which were 7.5 au, 15 au, and 30 au, were achieved by preparing a liquid dilution of MV that ranged from 1:1 to 1:4 and then diluting MV saturated vapor to 0.75 ± 0.12%, 1.66 ± 0.26%, and 3.13 ± 0.41%, respectively.

During all imaging preparations, 12 blocks of odorant trials (4 odorants × 3 concentrations) were presented to each subject via a manifold that was positioned ~1 cm in front of the nose. Each odor block consisted of 4, 16-sec trials that were presented at 60 sec inter-trial intervals (ITIs). Several blocks of blank (no odorant) trials (2–4 trials/block, 16 sec/trial, 60 sec ITIs) were also presented, and were then averaged together offline and subtracted from each odorant trial to correct for photobleaching. The 4 blank-subtracted odorant trials per block were averaged together to improve the signal-to-noise ratio of the data.

### Quantification and analysis of odorant-evoked optical signals

Imaging data were extracted and quantified after the manner of 27,86,87. Data were processed and analyzed in Neuroplex, Matlab, and SPSS, and were subsequently plotted in SigmaPlot, Matlab, and Origin.

Peak odorant-evoked response maps were generated by subtracting the average of 7 frames that were collected immediately prior to stimulus onset from the average of 7 frames that were approximately centered on the peak trace inflection. spH provides a relatively slow, cumulative signal, causing the peak response across most glomeruli to occur around the time of odorant offset, so frames 78–84 were used for this subtraction (see Fig. 1D,E and I for examples of peak subtractions). We then performed 3 more subtractions and generated a time course of pre-peak response maps that were evoked throughout each odorant presentation (see Fig. 2A,B for stimulus diagrams showing all subtractions). Specifically, we subtracted the average of 7 pre-odorant baseline frames from the average of frames 1) 36–42, 2) 50–56, and 3) 64–70. All difference maps were spatially filtered to separate discrete odorant-evoked spH signals from broad changes in tissue reflectance.

Putative glomerular regions of interest (ROIs) were first identified in the peak, spatially high-pass filtered difference maps, and were then confirmed through a statistical thresholding criterion^81^,^83^. If the mean evoked change in fluorescence (ΔF) across repeated trials was more than 3 standard errors greater than 0 for a glomerular ROI, then it was considered to be a response. The raw data set for the results that are summarized by Figs 1, 2, 3 included 369 glomerular ROIs from 8 unmanipulated males and 679 glomerular ROIs from 11 unmanipulated females. The raw data set for the results that are summarized by Figs 4, 5, 6 included 718 ROIs from 12 Sham-males, 783 ROIs from 10 Gnx-males, 852 ROIs from 11 Sham-females, and 604 ROIs from 10 Gnx-females.

The average number of glomerular responses contributing to each odor representation during each response time bin were quantified in all 12 spatially high-pass-filtered odor maps from each subject. We also calculated peak odorant-evoked ΔFs for all glomerular ROIs in each subject. Parametric tests, including factorial ANOVAs and *t* tests, were used to evaluate group differences in central tendencies based on means from individual subjects. In these analyses, odorant (BA, MV, 2HEX, and 2M2B), concentration (7.5 au, 15 au, and 30 au), and response time bin (frames 36–42, 50–56, 64–70, and 78–84) were used as within-subjects factors and sex (male and female) and surgical treatment (Sham and Gnx) were used as between-subjects factors.

To perform odorant response selectivity analyses, each individual glomerulus was identified as receiving input from OSNs that were stimulated by 1, 2, 3, or 4 odorants in the panel^83^, with lower numbers indicating relatively high odorant response selectivity and higher numbers indicating relatively low odorant response selectivity. These data were pooled across glomeruli and analyzed via ANOVAs and *t* tests that included sex and surgical treatment as between-groups factors.

To evaluate the time course of odorant-evoked nerve output, particularly during the relatively early, pre-peak part of the responses, traces from all glomerular ROIs were exported through custom software in Matlab. These data were extracted from trials corresponding to the moderate concentration (15 au) of all 4 odorants in the panel. Each individual trace, which represented a single glomerulus’ fluorescence throughout the length of an entire trial, was normalized relative to its own minimum and maximum values. We used the normalized traces to quantify the amount of time that it took for each glomerulus to reach its peak response magnitude. The latency to peak onset was calculated as time in sec and was constrained to the frame range corresponding to 2–9 sec after odorant onset. The mean latency to peak onset across each odor map was separately calculated for each mouse by averaging peak onset latencies across glomeruli within each odorant. These data were analyzed via ANOVAs that included sex and surgical treatment as between-subjects factors and odorant as a within-subjects factor. Additional non-parametric tests, including Kolmogorov-Smirnov (K-S) and Mann-Whitney (M-W) tests, were used to evaluate differences in latencies that were pooled across distributions of glomeruli.

To quantify differences in overall odor representations for each individual mouse, the pairwise Euclidean distances (EDs) between BA-, MV-, 2HEX-, and 2M2B-evoked glomerular response maps were calculated on a frame-by-frame basis. Differences among odorant-evoked glomerular response maps were quantified as EDs in *N*-dimensional vector space, where *N* was equivalent to the total number of glomerular ROIs identified across all 4 odorants in each mouse^27^. Each mouse’s ROI array consisted of all ROIs (ROI_1_-ROI_*N*_) that were identified as glomeruli receiving synaptic input across all 4 odorants in our test panel. For example, a hypothetical array might have included 80 ROIs that were identified across both olfactory bulbs and all 4 odorants, but maybe only 20 of those ROIs were identified as glomeruli receiving 2HEX-evoked input while the remaining 60 were identified as receiving input that was evoked by 1 or more of the 3 remaining odorants in the panel. 2HEX would then be represented by an array of ROIs that includes 20 “active” glomeruli that are receiving odorant-evoked OSN input and 80 “inactive” (response of zero) glomeruli that are not receiving odorant-evoked OSN input. ED calculations for each odor pair were performed on normalized data in which the fluorescence values were normalized relative to the maximum fluorescence value across all frames, ROIs, and both odorants within each odor pair. These data were analyzed via factorial ANOVAs that included sex and surgical treatment as between-subjects factors and also included odor pair (6; BA-MV, BA-2HEX, BA-2M2B, MV-2HEX, MV-2M2B, & 2HEX-2M2B) and frame number (64; frames 28–91, which correspond to 0–9 sec relative to odorant onset) as within-subjects factors. Additionally, a subset of subjects in the gonadectomy-imaging experiment (*N* = 18) were presented with 15 au ethyl valerate (EV), which is an ester that differs in functional group, but is highly similar to MV (another ester that was included in our 4 × 3 odor panel). The ED between EV- and MV-evoked odor maps was also quantified in this subset of mice and analyzed accordingly.

## Additional Information

**How to cite this article:** Kass, M. D. *et al*. Differences in peripheral sensory input to the olfactory bulb between male and female mice. *Sci. Rep.* **7**, 45851; doi: 10.1038/srep45851 (2017).

**Publisher's note:** Springer Nature remains neutral with regard to jurisdictional claims in published maps and institutional affiliations.

## Supplementary Material

Supplementary Information

## References

[b1] Dalton P., Doolittle N. & Breslin P. A. S. Gender-specific induction of enhanced sensitivity to odors. Nature Neuroscience 5, 199–200 (2002). 11865309 10.1038/nn803

[b2] Xu P. S., Lee D. & Holy T. E. Experience-Dependent Plasticity Drives Individual Differences in Pheromone-Sensing Neurons. Neuron 91, 878–892 (2016). 27537487 10.1016/j.neuron.2016.07.034 PMC5003430

[b3] Dorries K. M., Schmidt H. J., Beauchamp G. K. & Wysocki C. J. Changes in sensitivity to the odor of androstenone during adolescence. Dev Psychobiol 22, 423–435 (1989). 2759357 10.1002/dev.420220502

[b4] Hummel T., Kobal G., Gudziol H. & Mackay-Sim A. Normative data for the “Sniffin’ Sticks” including tests of odor identification, odor discrimination, and olfactory thresholds: an upgrade based on a group of more than 3,000 subjects. Eur Arch Otorhinolaryngol 264, 237–243 (2007). 17021776 10.1007/s00405-006-0173-0

[b5] Segal N. L., Topolski T. D., Wilson S. M., Brown K. W. & Araki L. Twin Analysis of Odor Identification and Perception. Physiology & Behavior 57, 605–609 (1995). 7538679 10.1016/0031-9384(94)00328-3

[b6] Koelega H. S. & Koster E. P. Some experiments on sex differences in odor perception. Annals of the New York Academy of Sciences 237, 234–246 (1974). 4528891 10.1111/j.1749-6632.1974.tb49859.x

[b7] Ohla K. & Lundstrom J. N. Sex differences in chemosensation: sensory or emotional? Front Hum Neurosci 7, 607 (2013). 24133429 10.3389/fnhum.2013.00607 PMC3783851

[b8] Wesson D. W., Keller M., Douhard Q., Baum M. J. & Bakker J. Enhanced urinary odor discrimination in female aromatase knockout (ArKO) mice. Horm Behav 49, 580–586 (2006). 16448653 10.1016/j.yhbeh.2005.12.013 PMC2263132

[b9] Doty R. L. & Cameron E. L. Sex differences and reproductive hormone influences on human odor perception. Physiol Behav 97, 213–228 (2009). 19272398 10.1016/j.physbeh.2009.02.032 PMC2693767

[b10] Baum M. J. & Keverne E. B. Sex difference in attraction thresholds for volatile odors from male and estrous female mouse urine. Horm Behav 41, 213–219 (2002). 11855906 10.1006/hbeh.2001.1749

[b11] Cometto-Muniz J. E. & Abraham M. H. Human olfactory detection of homologous n-alcohols measured via concentration-response functions. Pharmacol Biochem Behav 89, 279–291 (2008). 18258288 10.1016/j.pbb.2007.12.023 PMC2323909

[b12] Kobal G. . A threshold-like measure for the assessment of olfactory sensitivity: the “random” procedure. Eur Arch Otorhinolaryngol 258, 168–172 (2001). 11407447 10.1007/s004050100328

[b13] Pierman S., Douhard Q., Balthazart J., Baum M. J. & Bakker J. Attraction thresholds and sex discrimination of urinary odorants in male and female aromatase knockout (ArKO) mice. Horm Behav 49, 96–104 (2006). 15961088 10.1016/j.yhbeh.2005.05.007

[b14] Sorwell K. G., Wesson D. W. & Baum M. J. Sexually dimorphic enhancement by estradiol of male urinary odor detection thresholds in mice. Behav Neurosci 122, 788–793 (2008). 18729632 10.1037/0735-7044.122.4.788 PMC2866425

[b15] Doty R. L., Applebaum S., Zusho H. & Settle R. G. Sex differences in odor identification ability: a cross-cultural analysis. Neuropsychologia 23, 667–672 (1985). 4058710 10.1016/0028-3932(85)90067-3

[b16] Doty R. L. . Smell identification ability: changes with age. Science 226, 1441–1443 (1984). 6505700 10.1126/science.6505700

[b17] Doty R. L. & Ferguson-Segall M. Influence of adult castration on the olfactory sensitivity of the male rat: a signal detection analysis. Behav Neurosci 103, 691–694 (1989). 2736075 10.1037//0735-7044.103.3.691

[b18] Good P. R., Geary N. & Engen T. The effect of estrogen on odor detection. Chem Senses 2, 46–50 (1976).

[b19] Koster E. P. Olfactory sensitivity and ovulatory cycle duration. Olfactologia 1, 43–51 (1968).

[b20] Mair R. G., Bouffard J. A., Engen T. & Morton T. H. Olfactory sensitivity during the menstrual cycle. Sens Processes 2, 90–98 (1978). 715472

[b21] Pietras R. J. & Moulton D. G. Hormonal influences on odor detection in rats: changes associated with the estrous cycle, pseudopregnancy, ovariectomy, and administration of testosterone propionate. Physiol Behav 12, 475–491 (1974). 4820142 10.1016/0031-9384(74)90125-5

[b22] Caruso S. . Prospective study evaluating olfactometric and rhinomanometric outcomes in postmenopausal women on 1 mg 17beta-estradiol and 2 mg drospirenone HT. Menopause 15, 967–972 (2008). 18551084 10.1097/gme.0b013e31816be973

[b23] Deems D. A. . Smell and taste disorders, a study of 750 patients from the University of Pennsylvania Smell and Taste Center. Arch Otolaryngol Head Neck Surg 117, 519–528 (1991). 2021470 10.1001/archotol.1991.01870170065015

[b24] Dillon T. S., Fox L. C., Han C. & Linster C. 17beta-estradiol enhances memory duration in the main olfactory bulb in CD-1 mice. Behav Neurosci 127, 923–931 (2013). 24341716 10.1037/a0034839 PMC4112518

[b25] Dhong H. J., Chung S. K. & Doty R. L. Estrogen protects against 3-methylindole-induced olfactory loss. Brain Res 824, 312–315 (1999). 10196466 10.1016/s0006-8993(99)01241-x

[b26] Dias B. G. & Ressler K. J. Parental olfactory experience influences behavior and neural structure in subsequent generations. Nat Neurosci 17, 89–96 (2014). 24292232 10.1038/nn.3594 PMC3923835

[b27] Kass M. D., Rosenthal M. C., Pottackal J. & McGann J. P. Fear learning enhances neural responses to threat-predictive sensory stimuli. Science 342, 1389–1392 (2013). 24337299 10.1126/science.1244916 PMC4011636

[b28] Stowers L. & Liberles S. D. State-dependent responses to sex pheromones in mouse. Curr Opin Neurobiol 38, 74–79 (2016). 27093585 10.1016/j.conb.2016.04.001 PMC4921285

[b29] Fast C. D. & McGann J. P. Amygdalar gating of early sensory processing through interactions with locus coeruleus. *J Neurosci* In Press (2017). 10.1523/JNEUROSCI.2797-16.2017 PMC5354340 28188216

[b30] Barni T. . Sex steroids and odorants modulate gonadotropin-releasing hormone secretion in primary cultures of human olfactory cells. J Clin Endocrinol Metab 84, 4266–4273 (1999). 10566683 10.1210/jcem.84.11.6150

[b31] Nathan B. P., Tonsor M. & Struble R. G. Acute responses to estradiol replacement in the olfactory system of apoE-deficient and wild-type mice. Brain Res 1343, 66–74 (2010). 20447382 10.1016/j.brainres.2010.04.070 PMC2932439

[b32] Nathan B. P., Tonsor M. & Struble R. G. Long-term effects of estradiol replacement in the olfactory system. Exp Neurol 237, 1–7 (2012). 22691461 10.1016/j.expneurol.2012.06.001 PMC3418380

[b33] Cherian S., Wai Lam Y., McDaniels I., Struziak M. & Delay R. J. Estradiol rapidly modulates odor responses in mouse vomeronasal sensory neurons. Neuroscience 269, 43–58 (2014). 24680884 10.1016/j.neuroscience.2014.03.011 PMC4270699

[b34] Dey S. . Cyclic Regulation of Sensory Perception by a Female Hormone Alters Behavior. Cell 161, 1334–1344 (2015). 26046438 10.1016/j.cell.2015.04.052 PMC4501503

[b35] Bozza T., McGann J. P., Mombaerts P. & Wachowiak M. *In vivo* imaging of neuronal activity by targeted expression of a genetically encoded probe in the mouse. Neuron 42, 9–21 (2004). 15066261 10.1016/s0896-6273(04)00144-8

[b36] Spors H. & Grinvald A. Spatio-temporal dynamics of odor representations in the mammalian olfactory bulb. Neuron 34, 301–315 (2002). 11970871 10.1016/s0896-6273(02)00644-x

[b37] Smear M., Shusterman R., O’Connor R., Bozza T. & Rinberg D. Perception of sniff phase in mouse olfaction. Nature 479, 397–400 (2011). 21993623 10.1038/nature10521

[b38] Spors H., Wachowiak M., Cohen L. B. & Friedrich R. W. Temporal dynamics and latency patterns of receptor neuron input to the olfactory bulb. J Neurosci 26, 1247–1259 (2006). 16436612 10.1523/JNEUROSCI.3100-05.2006 PMC6674558

[b39] Kass M. D., Moberly A. H. & McGann J. P. Spatiotemporal alterations in primary odorant representations in olfactory marker protein knockout mice. PLoS One 8, e61431 (2013). 23630588 10.1371/journal.pone.0061431 PMC3632605

[b40] Lee A. C., He J. & Ma M. Olfactory marker protein is critical for functional maturation of olfactory sensory neurons and development of mother preference. J Neurosci 31, 2974–2982 (2011). 21414919 10.1523/JNEUROSCI.5067-10.2011 PMC3084592

[b41] Cury K. M. & Uchida N. Robust odor coding via inhalation-coupled transient activity in the mammalian olfactory bulb. Neuron 68, 570–585 (2010). 21040855 10.1016/j.neuron.2010.09.040

[b42] Uchida N. & Mainen Z. F. Speed and accuracy of olfactory discrimination in the rat. Nat Neurosci 6, 1224–1229 (2003). 14566341 10.1038/nn1142

[b43] Wesson D. W., Carey R. M., Verhagen J. V. & Wachowiak M. Rapid encoding and perception of novel odors in the rat. PLoS Biol 6, e82 (2008). 18399719 10.1371/journal.pbio.0060082 PMC2288628

[b44] Linster C. . Perceptual correlates of neural representations evoked by odorant enantiomers. J Neurosci 21, 9837–9843 (2001). 11739591 10.1523/JNEUROSCI.21-24-09837.2001 PMC6763025

[b45] Youngentob S. L., Johnson B. A., Leon M., Sheehe P. R. & Kent P. F. Predicting odorant quality perceptions from multidimensional scaling of olfactory bulb glomerular activity patterns. Behav Neurosci 120, 1337–1345 (2006). 17201479 10.1037/0735-7044.120.6.1337 PMC2222860

[b46] Kimchi T., Xu J. & Dulac C. A functional circuit underlying male sexual behaviour in the female mouse brain. Nature 448, 1009–1014 (2007). 17676034 10.1038/nature06089

[b47] Stowers L. & Logan D. W. Sexual dimorphism in olfactory signaling. Curr Opin Neurobiol 20, 770–775 (2010). 20833534 10.1016/j.conb.2010.08.015 PMC3005963

[b48] Oliva A. M. . Toward a mouse neuroethology in the laboratory environment. PLoS One 5, e11359 (2010). 20613876 10.1371/journal.pone.0011359 PMC2894054

[b49] Rinberg D., Koulakov A. & Gelperin A. Speed-accuracy tradeoff in olfaction. Neuron 51, 351–358 (2006). 16880129 10.1016/j.neuron.2006.07.013

[b50] Kawagishi K. . Stereological quantification of olfactory receptor neurons in mice. Neuroscience 272, 29–33 (2014). 24797329 10.1016/j.neuroscience.2014.04.050

[b51] Buiakova O. I. . Olfactory marker protein (OMP) gene deletion causes altered physiological activity of olfactory sensory neurons. Proc Natl Acad Sci USA 93, 9858–9863 (1996). 8790421 10.1073/pnas.93.18.9858 PMC38519

[b52] Tsim T. Y., Wong E. Y., Leung M. S. & Wong C. C. Expression of axon guidance molecules and their related genes during development and sexual differentiation of the olfactory bulb in rats. Neuroscience 123, 951–965 (2004). 14751288 10.1016/j.neuroscience.2003.10.024

[b53] Labhsetwar A. P. Pituitary levels of FSH and LH at various intervals after ovariectomy in the rat. J Reprod Fertil 18, 531–533 (1969). 5788223 10.1530/jrf.0.0180531

[b54] Yen S. S. & Tsai C. C. The effect of ovariectomy on gonadotropin release. J Clin Invest 50, 1149–1153 (1971). 5552412 10.1172/JCI106587 PMC292038

[b55] Shiao M. S. . Transcriptomes of mouse olfactory epithelium reveal sexual differences in odorant detection. Genome Biol Evol 4, 703–712 (2012). 22511034 10.1093/gbe/evs039 PMC3381674

[b56] Lupo C., Lodi L., Canonaco M., Valenti A. & Dessi-Fulgheri F. Testosterone metabolism in the olfactory epithelium of intact and castrated male rats. Neurosci Lett 69, 259–262 (1986). 3763055 10.1016/0304-3940(86)90490-8

[b57] Zhou X. . High abundance of testosterone and salivary androgen-binding protein in the lateral nasal gland of male mice. J Steroid Biochem Mol Biol 117, 81–86 (2009). 19524040 10.1016/j.jsbmb.2009.06.002 PMC2749885

[b58] McGann J. P. Presynaptic inhibition of olfactory sensory neurons: new mechanisms and potential functions. Chem Senses 38, 459–474 (2013). 23761680 10.1093/chemse/bjt018 PMC3685425

[b59] Kiyokage E. . Molecular identity of periglomerular and short axon cells. J Neurosci 30, 1185–1196 (2010). 20089927 10.1523/JNEUROSCI.3497-09.2010 PMC3718026

[b60] Kosaka T. & Kosaka K. Tyrosine hydroxylase-positive GABAergic juxtaglomerular neurons are the main source of the interglomerular connections in the mouse main olfactory bulb. Neurosci Res 60, 349–354 (2008). 18206259 10.1016/j.neures.2007.11.012

[b61] Li T. Y. & Wu C. H. [Changes in the Amount of Gamma-Aminobutyric Acid in the Male Rat Brain after Castration]. Sheng Li Xue Bao 27, 1–4 (1964). 14184582

[b62] Maggi A. & Perez J. Estrogen-induced up-regulation of gamma-aminobutyric acid receptors in the CNS of rodents. J Neurochem 47, 1793–1797 (1986). 3021904 10.1111/j.1471-4159.1986.tb13090.x

[b63] Saad A. F. The effect of ovariectomy on the gamma-aminobutyric acid content in the cerebral hemispheres of young rats. J Pharm Pharmacol 22, 307–308 (1970). 4392576 10.1111/j.2042-7158.1970.tb08526.x

[b64] Wallis C. J. & Luttge W. G. INfluence of estrogen and progesterone on glutamic acid decarboxylase activity in discrete regions of rat brain. J Neurochem 34, 609–613 (1980). 7354335 10.1111/j.1471-4159.1980.tb11187.x

[b65] Weiland N. G. Glutamic acid decarboxylase messenger ribonucleic acid is regulated by estradiol and progesterone in the hippocampus. Endocrinology 131, 2697–2702 (1992). 1446611 10.1210/endo.131.6.1446611

[b66] Dluzen D. E., Park J. H. & Kim K. Modulation of olfactory bulb tyrosine hydroxylase and catecholamine transporter mRNA by estrogen. Brain Res Mol Brain Res 108, 121–128 (2002). 12480184 10.1016/s0169-328x(02)00520-x

[b67] Gao X. & Dluzen D. E. Tamoxifen abolishes estrogen’s neuroprotective effect upon methamphetamine neurotoxicity of the nigrostriatal dopaminergic system. Neuroscience 103, 385–394 (2001). 11246153 10.1016/s0306-4522(01)00014-8

[b68] Merchenthaler I., Lane M. V., Numan S. & Dellovade T. L. Distribution of estrogen receptor alpha and beta in the mouse central nervous system: *in vivo* autoradiographic and immunocytochemical analyses. J Comp Neurol 473, 270–291 (2004). 15101093 10.1002/cne.20128

[b69] Mitra S. W. . Immunolocalization of estrogen receptor beta in the mouse brain: comparison with estrogen receptor alpha. Endocrinology 144, 2055–2067 (2003). 12697714 10.1210/en.2002-221069

[b70] Horvath T. L. & Wikler K. C. Aromatase in developing sensory systems of the rat brain. J Neuroendocrinol 11, 77–84 (1999). 10048462 10.1046/j.1365-2826.1999.00285.x

[b71] Hoyk Z. . Aromatase and estrogen receptor beta expression in the rat olfactory bulb: Neuroestrogen action in the first relay station of the olfactory pathway? Acta Neurobiol Exp (Wars) 74, 1–14 (2014). 24718039 10.55782/ane-2014-1967

[b72] Gomez C. . Sex differences in catechol contents in the olfactory bulb of control and unilaterally deprived rats. Eur J Neurosci 25, 1517–1528 (2007). 17425578 10.1111/j.1460-9568.2007.05407.x

[b73] Alizadeh R. . Gender and age related changes in number of dopaminergic neurons in adult human olfactory bulb. J Chem Neuroanat 69, 1–6 (2015). 26212581 10.1016/j.jchemneu.2015.07.003

[b74] Ennis M. . Dopamine D2 receptor-mediated presynaptic inhibition of olfactory nerve terminals. J Neurophysiol 86, 2986–2997 (2001). 11731555 10.1152/jn.2001.86.6.2986

[b75] Guillamon A. & Segovia, S. Sex differences in the vomeronasal system. Brain Res Bull 44, 377–382 (1997). 9370202 10.1016/s0361-9230(97)00217-7

[b76] Kang N., Baum M. J. & Cherry J. A. A direct main olfactory bulb projection to the ‘vomeronasal’ amygdala in female mice selectively responds to volatile pheromones from males. Eur J Neurosci 29, 624–634 (2009). 19187265 10.1111/j.1460-9568.2009.06638.x PMC2669936

[b77] Schaefer M. L., Young D. A. & Restrepo D. Olfactory fingerprints for major histocompatibility complex-determined body odors. J Neurosci 21, 2481–2487 (2001). 11264322 10.1523/JNEUROSCI.21-07-02481.2001 PMC6762408

[b78] Veyrac A., Wang G., Baum M. J. & Bakker J. The main and accessory olfactory systems of female mice are activated differentially by dominant versus subordinate male urinary odors. Brain Res 1402, 20–29 (2011). 21683943 10.1016/j.brainres.2011.05.035 PMC3155078

[b79] Waters P., Woodley S. K. & Baum M. J. Sex difference in the distribution and size of glomeruli in the ferret’s main olfactory bulb. Neurosci Lett 381, 237–241 (2005). 15896476 10.1016/j.neulet.2005.02.042

[b80] Woodley S. K. & Baum M. J. Differential activation of glomeruli in the ferret’s main olfactory bulb by anal scent gland odours from males and females: an early step in mate identification. Eur J Neurosci 20, 1025–1032 (2004). 15305871 10.1111/j.1460-9568.2004.03571.x PMC1237011

[b81] Czarnecki L. A. . *In vivo* visualization of olfactory pathophysiology induced by intranasal cadmium instillation in mice. Neurotoxicology 32, 441–449 (2011). 21443902 10.1016/j.neuro.2011.03.007 PMC3951954

[b82] Caligioni C. S. Assessing reproductive status/stages in mice. *Curr Protoc Neurosci* Appendix 4, Appendix 4I (2009). 10.1002/0471142301.nsa04is48 PMC2755182 19575469

[b83] Kass M. D., Pottackal J., Turkel D. J. & McGann J. P. Changes in the neural representation of odorants after olfactory deprivation in the adult mouse olfactory bulb. Chem Senses 38, 77–89 (2013). 23125347 10.1093/chemse/bjs081 PMC3522516

[b84] Moberly A. H. . Intranasal exposure to manganese disrupts neurotransmitter release from glutamatergic synapses in the central nervous system *in vivo*. Neurotoxicology 33, 996–1004 (2012). 22542936 10.1016/j.neuro.2012.04.014 PMC3432160

[b85] Czarnecki L. A. . Functional rehabilitation of cadmium-induced neurotoxicity despite persistent peripheral pathophysiology in the olfactory system. Toxicol Sci 126, 534–544 (2012). 22287023 10.1093/toxsci/kfs030 PMC3307613

[b86] Kass M. D., Moberly A. H., Rosenthal M. C., Guang S. A. & McGann J. P. Odor-specific, olfactory marker protein-mediated sparsening of primary olfactory input to the brain after odor exposure. J Neurosci 33, 6594–6602 (2013). 23575856 10.1523/JNEUROSCI.1442-12.2013 PMC3865540

[b87] Kass M. D., Guang S. A., Moberly A. H. & McGann J. P. Changes in Olfactory Sensory Neuron Physiology and Olfactory Perceptual Learning After Odorant Exposure in Adult Mice. Chem Senses 41, 123–133 (2016). 26514410 10.1093/chemse/bjv065 PMC4751239

