# Supplementary material for: Differences in peripheral sensory input to the olfactory bulb between male and female mice
Source: Sci Rep. 2017 Apr 26;7:45851. doi: 10.1038/srep45851 (PMC5405412; doi:10.1038/srep45851)
Supplement: Supplementary Information [file srep45851-s1.pdf]

Differences in peripheral sensory input to the olfactory bulb between male and female mice

Supplementary Information

Marley D. Kass, Lindsey A. Czarnecki, Andrew H. Moberly, and John P. McGann

Behavioral & Systems Neuroscience Section
Department of Psychology
Rutgers, The State University of New Jersey
152 Frelinghuysen Road
Piscataway, NJ 08854

Corresponding author: John P. McGann, PhD
Department of Psychology
Rutgers University
152 Frelinghuysen Road
Piscataway, NJ 08854 USA
Email: john.mcgann@rutgers.edu

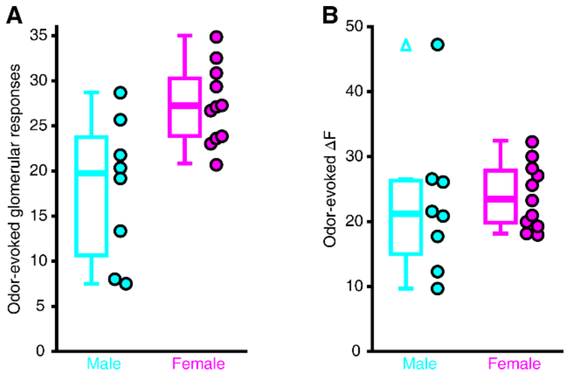

Supplementary Fig. S1: Individual variability in the number of glomeruli receiving odorant-evoked OSN input and peak response amplitudes in unmanipulated males and females. (A-B) Box plots showing the distributions of odorant-evoked glomerular responses **(A)** and odorant-evoked response amplitudes **(**ΔFs, **B)** for male and female subjects. Box, 25th-75th percentile; thick, solid line, median; whiskers, minimum and maximum; open triangles, outliers 1.5× the interquartile range. Individual subjects are represented by circles that are plotted immediately to the right of each distribution. **(A)** Odorant-evoked glomerular responses are plotted for the individual male (N = 8) and female (N = 11) subjects that contributed to the analyses summarized in Fig. 1C. Each individual subject is represented here by the average number of odorant-evoked glomerular responses that was observed across all 3 concentrations of all 4 odorants; Mann-Whitney U test, Z = -2.642, p = 0.008. **(B)** Odorant-evoked ΔFs from the individual male (N = 8) and female (N = 11) subjects that contributed to the analyses summarized in Fig. 1J. Each individual subject is represented here by the average odorant-evoked ΔF calculated across all 3 concentrations of all 4 odorants; Mann-Whitney U test, Z = -0.826, p = 0.409.

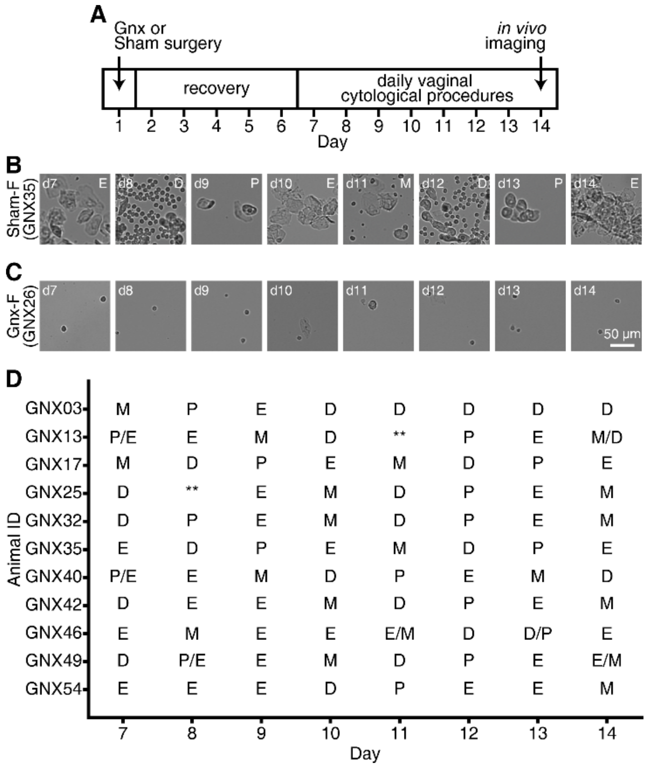

Supplementary Fig. S2: Procedure summary for the gonadectomy-imaging experiment. (A) Experimental timeline showing gonadectomy (Gnx) or sham-control (Sham) procedures (day 1), followed by daily vaginal cytological procedures (days 7-14), and finally *in vivo* optical imaging procedures (day 14). **(B-C)** Example images of unstained vaginal secretion from a Sham-female **(B,** GNX35**)** and a Gnx-female **(C,** GNX26**)** across 8 consecutive days (d7-d14). P, proestrus; E, estrus; M, metestrus; D, diestrus. **(D)** The estrous cycle of all 11 Sham-females is plotted across 8 consecutive days leading up to the optical imaging experiment. **unable to determine from sample. Note that subjects in the gonadectomy (GNX)-imaging experiment were identified in sequence as GNX_{01} through GNX_{NN}, regardless of sex or surgical group assignment.

Vaginal smear cytology[82] was used to determine the estrous cycle phases in Sham-females and to confirm the efficacy of ovariectomy in Gnx-females. Females were gently restrained, the vulva was cleaned with gauze soaked in saline, and the vagina was then gently flushed 5-7 times with a sterile saline solution. The final flush was collected back into the pipette

and then dispensed on a glass slide for cytological analysis via brightfield microscopy. Photographs of vaginal secretion were taken at a magnification of 10× with a Jenoptik MFcool Peltier-cooled CCD camera mounted on an Olympus BX41 microscope. Note that males underwent a similar "sham-smear" procedure to maintain equal treatment across all experimental groups. For the sham-smear procedure, males were gently restrained and the genital area was cleaned with saline solution and delicately prodded with a pipette.

The estrous cycle stages were identified in Sham-females based on the proportion of cell types that were observed in the vaginal secretion (Supplementary Fig. S2B). Overall, we observed approximately normal, 4-day cycles in Sham-females (Supplementary Fig. S2B and S2D). Macroanatomic manifestations of the estrous cycle[88,89] were also observed in Sham-females through visual inspection that was performed during the restraint that occurred immediately prior to performing vaginal smear procedures.

The density of cells in samples that were collected from Gnx-females was notably lower than that observed in samples from Sham-females (compare Supplementary Figs. S2B and S2C), and there was no change in the relative proportion of different cell types from day to day. In fact, very few cells were present in many of the Gnx-female samples (Supplementary Fig. S2C), which is consistent with observations from other groups[90]. While there were no day to day changes in the appearance of the vagina in Gnx-females, there was a striking difference in the appearance of the vaginal opening in Gnx-females relative to Sham-females. Consistent with other reports[90], the vaginal opening in Gnx-females was pale in coloration, very dry, and extremely narrow/closed.

The differences across days in the vaginal opening and vaginal smears that were observed in Sham-females, and the lack of such differences in Gnx-females, suggests that circulating

gonadal hormones were relatively unaffected by the sham-surgical procedures but successfully eliminated by gonadectomy.

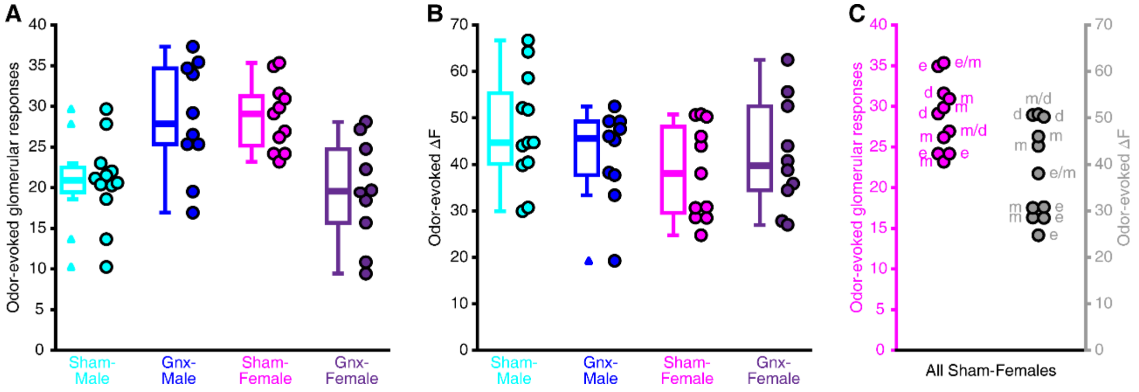

Supplementary Fig. S3: Odorant-evoked glomerular responses and response amplitudes from all individual subjects from sex × surgical treatment groups. (A-B) Box plots showing the distributions of odorant-evoked glomerular responses **(A)** and odorant-evoked response amplitudes (ΔFs, **B)** from sex × surgical treatment groups. Box, 25th-75th percentile; thick, solid line, median; whiskers, minimum and maximum; closed triangles, outliers 1.5× the interquartile range. Individual subjects are represented by circles plotted immediately to the right of each distribution. **(A)** Odorant-evoked glomerular responses are plotted for the individual Sham-male (N = 12), Gnx-male (N = 10), Sham-female (N = 11), and Gnx-female (N = 10) subjects that contributed to the analyses summarized in Fig. 4I-J. Each individual subject is represented here by the average number of odorant-evoked glomerular responses that was observed across all 3 concentrations of all 4 odorants; Kruskal-Wallis test across 4 groups, $\chi^2_{(df=3)} = 15.915$, $p = 0.001$. *Post hoc* Mann-Whitney tests between pairwise group comparisons: Sham-male versus Gnx-male, $Z = -2.243$, $p = 0.025$; Sham-male versus Sham-female, $Z = -3.386$, $p < 0.001$; Sham-male versus Gnx-female, non-significant, $Z = -0.593$, $p = 0.582$; Sham-female versus Gnx-female, $Z = -2.959$, $p = 0.002$; Sham-female versus Gnx-male, non-significant, $Z = -0.070$, $p = 0.973$; Gnx-female versus Gnx-male, $Z = -2.420$, $p = 0.015$. **(B)** Odorant-evoked ΔF from the individual subjects that contributed to the analyses summarized in Fig. 4M. Each individual subject is represented here by the average odorant-evoked ΔF calculated across all 3 concentrations of all 4

odorants; non-significant Kruskal-Wallis test across 4 groups, $\chi^2_{(df=3)} = 3.143$, $p = 0.370$. **(C)** The data that are plotted for the Sham-Female group in panels **A** and **B** are replotted with lettering to indicate the phase of the estrous cycle for each subject. The left *y*-axis is shown in magenta and corresponds with the mean number of odorant-evoked glomerular responses per subject, and the right *y*-axis is shown in grey and corresponds with the mean odorant-evoked ΔF per subject. p, proestrus; e, estrus; m, metestrus; d, diestrus.

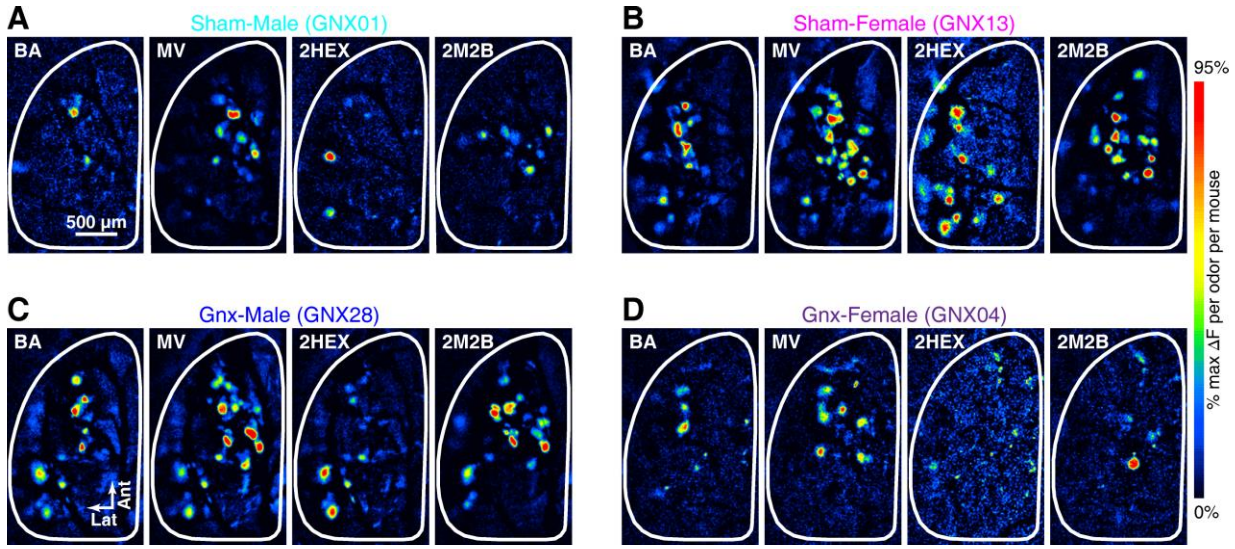

Supplementary Fig. S4: Glomerular response maps that were evoked by two esters, a ketone, and an aldehyde. (A-D) Additional pseudocolored difference maps from the representative Sham-male **(A,** GNX01**)**, Sham-female **(B,** GNX13**)**, Gnx-male **(C,** GNX28**)**, and Gnx-female **(D,** GNX04**)** subjects that are shown in Fig. 4A-D. The maps that are shown in Fig. 4A-D were evoked across a 4-fold range of BA concentrations, and thus illustrate the interactive effects of sex and surgical treatment on concentration-dependent glomerular recruitment. To further demonstrate that the interactive effects of sex and surgical treatment were observed in response to all of the odorants that we tested, the peak odorant-evoked glomerular response maps that were evoked by the 15 au concentration of two esters (BA and MV), a ketone (2HEX), and an aldehyde (2M2B) are shown here for each subject. Regardless of the odorant (or concentration, Fig. 4A-D) that was being presented, a larger number of olfactory bulb glomeruli received OSN synaptic input in Sham-females than in Sham-males (compare panels **A** and **B**). Interestingly, this sexually dimorphic activation of olfactory bulb glomeruli seemed to be reversed by gonadectomy because there were a larger number of odorant-evoked glomerular responses in Gnx-males than in Gnx-females (compare panels **C** and **D**). Thus, patterns of

glomeruli receiving odorant-evoked synaptic input in gonadectomized males and females are more similar to opposite-sex control animals than they are to same-sex control animals.

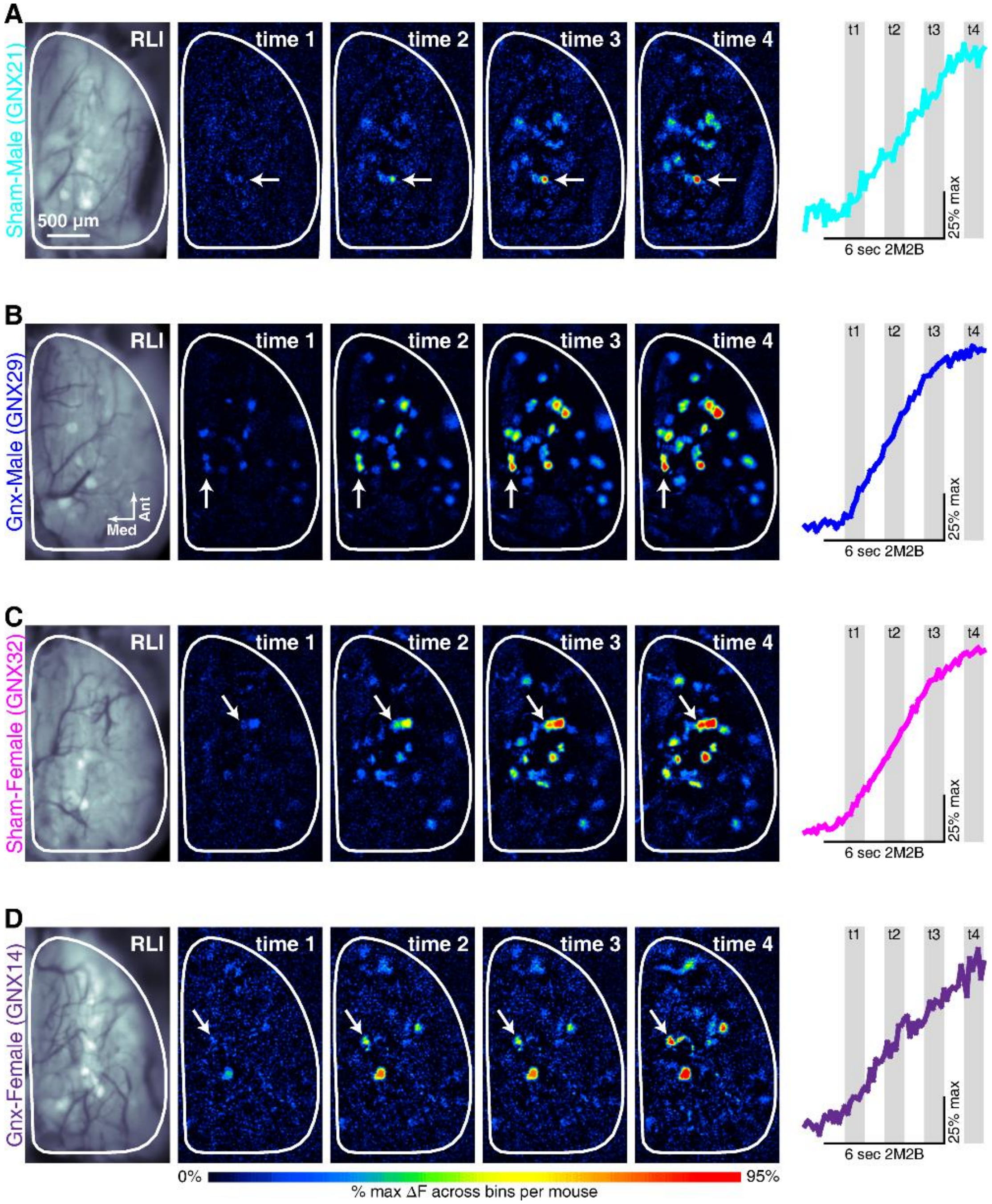

Supplementary Fig. S5: Time course of odorant-evoked glomerular response maps. (A-D) Each panel shows a resting light intensity (RLI) image through the cranial window along with pseudocolored difference maps that were measured during 4, sequential 1-sec time bins (time 1-4), from representative Sham-male **(A,** GNX21**)**, Gnx-male **(B,** GNX29**)**, Sham-female **(C,** GNX32**)**, and Gnx-female **(D,** GNX14**)** subjects. A timeline is shown in the right of each panel

to illustrate the 4, 1-sec time bins (shaded regions; t1-t4, response times 1-4) that were used to generate the example glomerular response maps in **A-D** and the analyses that are summarized in Fig. 4K. The example fluorescent records that are superimposed on each timeline correspond to the glomerular callouts (white arrows) on the difference maps in **A-D**, which are 4-trial block averages of 15 au 2M2B.

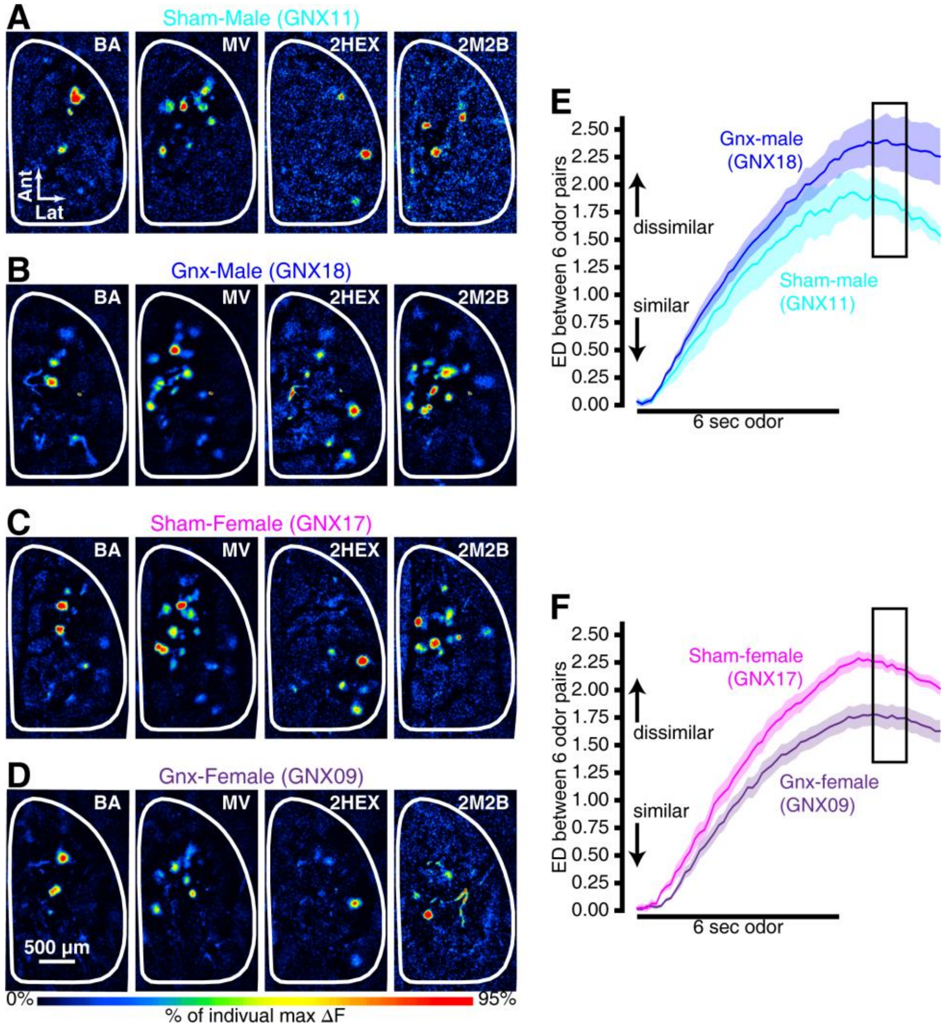

Supplementary Fig. S6: Pairs of odor maps become closer together in Euclidean space after gonadectomy in females, but further apart after gonadectomy in males. (A-D) BA-, MV-, 2HEX-, and 2M2B-evoked difference maps from representative Sham-male **(A,** GNX11**)**, Gnx-male **(B,** GNX18**)**, Sham-female **(C,** GNX17**)**, and Gnx-female **(D,** GNX09**)** subjects. **(E-F)** The mean±SEM Euclidean distance (ED) is calculated between all 6 odor pairs (BA-MV, BA-2HEX, BA-2M2B, MV-2HEX, MV-2M2B, AND 2HEX-2M2B) and across 64 consecutive frames (from time = 0-9 sec relative to odorant onset) for each representative subject shown in **A-D**. The black stimulus bar indicates the time of odorant presentations and the boxed region of the mean±SEM EDs notes the frames that were used to generate the difference maps in **A-D**. Odor

maps were more dissimilar from each other (i.e., further apart in Euclidean space) in the example Gnx-male than in the example Sham-male **(E)**. By contrast, odor maps were more similar to each other (i.e., closer together in Euclidean space) in the example Gnx-female than in the example Sham-female **(F)**.

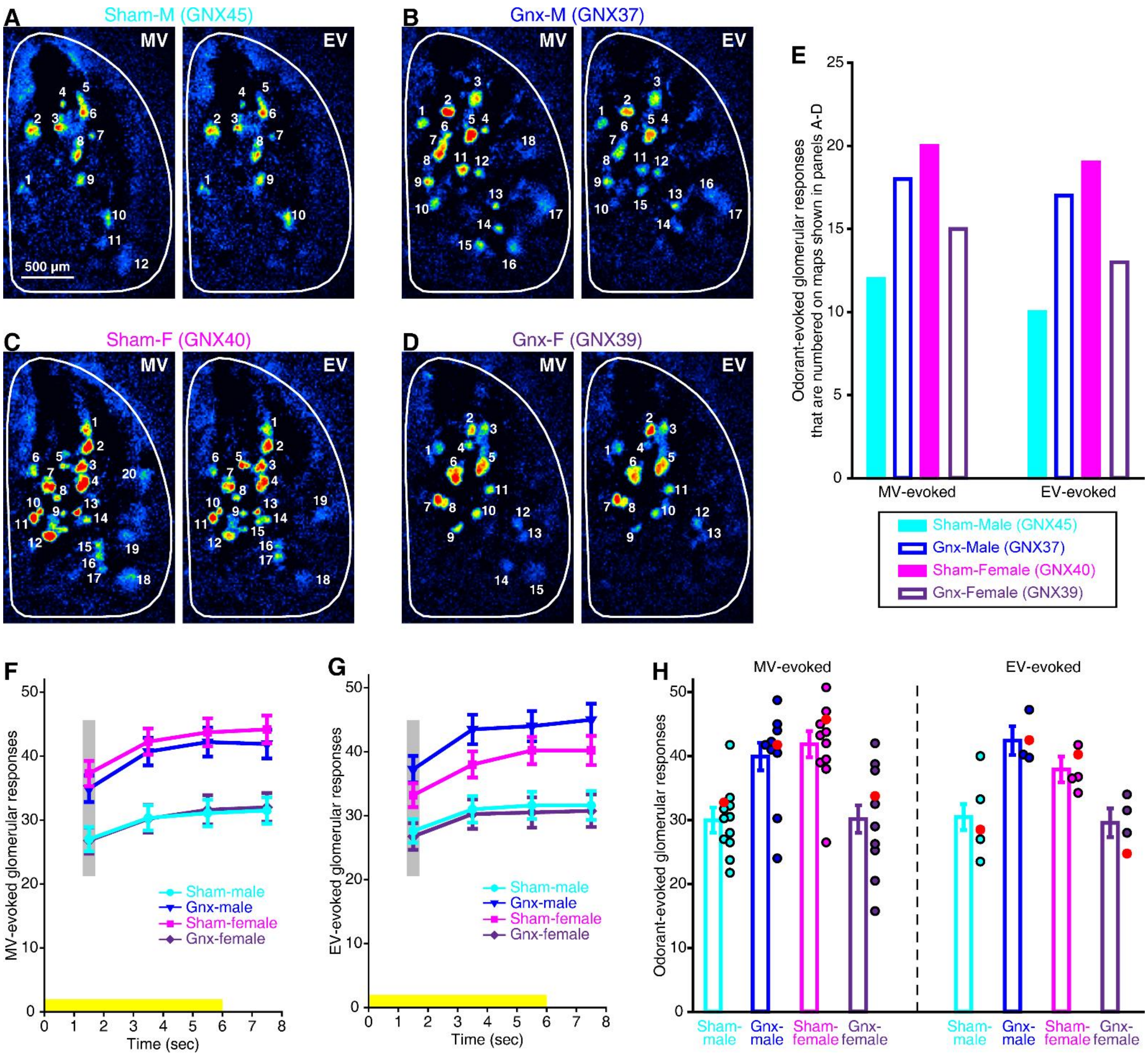

Supplementary Fig. S7: The interactive effects of sex and surgical treatment on the number of glomeruli receiving OSN synaptic input are observed in response to the presentation of chemically similar odorants. (A-D) The pairs of MV- and EV-evoked difference maps that are shown in Fig. 6C-F are enlarged here and are shown for the same Sham-male **(A,** GNX45**)**, Gnx-male **(B,** GNX37**)**, Sham-female **(C**, GNX40**)**, and Gnx-female **(D,** GNX39**)** subjects. Each pair of maps is scaled relative to the overall maximum across odorants, as specified in Fig. 6C-F. All glomerular regions of interest (ROIs) that are receiving odorant-evoked OSN input are numbered

separately (from ROI_1-ROI_N) for each individual difference map. **(E)** The glomerular ROIs that are numbered in **A-D** are separated by odorant and plotted for each individual subject. Note that these values are only used for demonstration purposes because they only reflect the number of odorant-evoked glomerular responses that are visible on the olfactory bulb that is being displayed, while the data were actually analyzed across both olfactory bulbs as shown in **F-H**. **(F-G)** The number of glomerular responses that was evoked by MV **(F)** and EV **(G)** is plotted as a function of time for each group. The difference maps that are shown in **A-D** correspond to the shaded response time bin, which occurred 1-2 sec into the stimulus presentation. The time of stimulus presentation is noted by the yellow bar. **(H)** The number of glomerular responses that was evoked by MV (left) and EV (right) is collapsed across all 4 response time bins for each group. Individual subjects are represented by circles that are plotted immediately to the right of each group mean. Red circles indicate the subjects whose difference maps are shown in **A-D**. The data shown in **F-H** are plotted as the mean±SEM.

The MV-evoked data that is shown here was included in the analyses that are summarized in Fig. 4 because it came from the entire study sample; Sham-male, N = 12, Gnx-male, N = 10, Sham-female, N = 11, and Gnx-female, N = 10. The EV-evoked data that is shown here was analyzed separately because only a subset of the subjects from that sample were presented with EV during their imaging preparations; Sham-male, N = 5, Gnx-male, N = 4, Sham-female, N = 5, and Gnx-female, N = 4. EV-evoked glomerular responses were analyzed via a sex (Male, Female) × surgical treatment (Sham, Gnx) × response time bin (time 1, time 2, time 3, time 4) mixed ANOVA, with sex and surgical treatment as between-subjects factors and response time bin as a within-subjects factor.

There was a significant interaction between sex and surgical treatment (Supplementary Fig. S7H, right panel; $F_{(3, 42)} = 5.452$, $p = 0.003$, $\eta_p^2 = 0.618$) such that Sham-females exhibited more EV-evoked glomerular responses than Sham-males ($F_{(1, 8)} = 5.550$, $p = 0.046$, $\eta_p^2 = 0.410$), while Gnx-females exhibited fewer glomerular responses than Gnx-males ($F_{(1, 6)} = 23.619$, $p = 0.003$, $\eta_p^2 = 0.797$). Gonadectomy thus resulted in opposite effects on EV-evoked glomerular responses in males and females, with the number of EV-evoked glomerular responses being reduced in Gnx-females relative to Sham-females ($F_{(1, 7)} = 12.574$, $p = 0.009$, $\eta_p^2 = 0.642$), but increased in Gnx-males relative to Sham-males ($F_{(1, 7)} = 11.277$, $p = 0.012$, $\eta_p^2 = 0.617$). The results from this analysis 1) replicate the findings from the analyses that were performed on the main data set (Fig. 4), which included 4 structurally- and perceptually-disparate odorants, and 2) extend those findings to a pair of structurally-similar odorants (which could potentially be relatively more challenging to discriminate).

Additional References

88 Byers, S. L., Wiles, M. V., Dunn, S. L. & Taft, R. A. Mouse estrous cycle identification tool and images. *PLoS One* **7**, e35538 (2012).

89 Champlin, A. K., Dorr, D. L. & Gates, A. H. Determining the stage of the estrous cycle in the mouse by the appearance of the vagina. *Biol Reprod* **8**, 491-494 (1973).

90 Ng, K. Y., Yong, J. & Chakraborty, T. R. Estrous cycle in ob/ob and ovariectomized female mice and its relation with estrogen and leptin. *Physiol Behav* **99**, 125-130 (2010).
